# Comparison of microbial signatures between paired faecal and rectal biopsy samples from healthy volunteers using next-generation sequencing and culturomics

**DOI:** 10.1186/s40168-022-01354-4

**Published:** 2022-10-14

**Authors:** Indrani Mukhopadhya, Jennifer C. Martin, Sophie Shaw, Aileen J. McKinley, Silvia W. Gratz, Karen P. Scott

**Affiliations:** 1 Gut Health Group, Rowett Institute, University of Aberdeen, Aberdeen, UK; 2 Centre for Genome Enabled Biology and Medicine, University of Aberdeen, Old Aberdeen, UK; 3 Current Address - All Wales Medical Genomics Service, Institute of Medical Genetics, University Hospital of Wales, Heath Park, Cardiff, UK; 4 Department of Surgery, Aberdeen Royal Infirmary Foresterhill, Aberdeen, UK

**Keywords:** Faecal microbiota, Mucosa-associated microbiota, Microbiome analysis, Next-generation sequencing, Anaerobic bacteria, Culture, SCFA

## Abstract

**Background:**

Faecal samples are frequently used to characterise the gut microbiota in health and disease, yet there is considerable debate about how representative faecal bacterial profiles are of the overall gut community. A particular concern is whether bacterial populations associated with the gut mucosa are properly represented in faecal samples, since these communities are considered critical in the aetiology of gastrointestinal diseases. In this study we compared the profiles of the faecal and mucosal microbiota from ten healthy volunteers using bacterial culturing (culturomics) and next-generation sequencing targeting the 16S ribosomal nucleic acid (rRNA) gene. Paired fresh rectal biopsies and faecal samples were processed under stringent anaerobic conditions to maintain the viability of the bacteria. Four different sample types were analysed: faecal (F), faecal homogenised (FHg), biopsy tissue (B) and biopsy wash (BW) samples.

**Results:**

There were no significant statistical differences in either bacterial richness or diversity between biopsy washes (BW) and faecal (F) or faecal homogenised (FHg) samples. Principal coordinates analysis of a Bray–Curtis distance matrix generated from sequence variant tables did not show distinct clustering between these samples (PERMANOVA; *p* = 0.972) but showed strong clustering of samples from individual donors. However, the rectal biopsy tissue (B) samples had a significantly altered bacterial signature with greater abundance of Proteobacteria and Acidobacteria compared to faecal (F) and faecal homogenised (FHg) samples. A total of 528 bacteria encompassing 92 distinct bacterial species were isolated and cultured from a subset of six volunteer samples (biopsy washes and faeces). This included isolation of 22 novel bacterial species. There was significant similarity between the bacterial species grown in anaerobic culture and those identified by 16S rRNA gene sequencing (Spearman correlation; rho = 0.548, *p* = 0.001).

**Conclusion:**

This study showed that the bacterial profiles of paired faecal and rectal biopsy wash samples were very similar, validating the use of faecal samples as a convenient surrogate for rectal biopsy-associated microbiota. Anaerobic bacterial culture results showed similar taxonomic patterns to the amplicon sequence analysis disproving the dogma that culture-based methods do not reflect findings of molecular assessments of gut bacterial composition.

Video abstract

**Supplementary Information:**

The online version contains supplementary material available at 10.1186/s40168-022-01354-4.

## Background

The gut microbiota is a complex entity comprising of myriad species of bacteria, archaea, fungi and viruses that are critical in homeostasis and disease [1]. There is variability in the microbial composition in different micro-environments across different segments of the gut [2]. Within a particular anatomical site, differences have also been noted amongst the more entrenched bacterial species adherent to the mucosa and those bacteria residing within the lumen [2]. Due to the proximity of the mucosa-associated bacteria to the host innate system, these adherent bacteria are postulated to play a key role in the aetiopathogenesis of various gastrointestinal diseases spanning from inflammatory bowel disease to colorectal cancer to necrotizing enterocolitis [3–6]. The luminal stream of bacteria on the other hand is considered to be affected by dietary changes and more likely to be represented in the extruded faecal samples [7].

Faecal samples have been commonly used as surrogate representatives of bacterial communities residing in the colon but there is debate on whether faecal samples accurately represent the mucosa-associated bacteria [8–12]. Most of the large-scale human health-related gut microbiome assessments including the Human Microbiome Project and MetaHIT have focussed on faecal microbiota due to the ease of access of samples without the need for invasive endoscopic procedures [13, 14]. It is therefore imperative that this wealth of information from faecal microbiota studies can be extrapolated accurately. There have been several studies comparing faecal and mucosal bacterial profiles with traditional culture methods and subsequently with modern sequencing techniques, but varying methodologies of sample collection and processing have been employed making a comparative analysis of such studies challenging [12, 15–17]. The yield of information has been vastly improved by moving away from the traditional culture-based techniques to next generation sequencing and metagenomic assessment of ‘unculturable’ gut bacteria [18]. Culture-based methods can also result in under-representation of low-abundance, difficult to grow bacterial species which is mitigated with the new techniques. However, the results of high throughput sequencing of the human colonic bacteria need to be interpreted with caution as the findings are not only dependent on the experimental design but can be influenced by the type of sample, the number of sequence reads, the DNA extraction method and sequencing primers utilized [19].

There has been a recent resurgence of culturing techniques with a specific focus on anaerobic conditions for collection and assessment of the gut bacterial species which has narrowed the gap with the more modern assessment of the enteric microbiome by next generation sequencing [20, 21]. The greatest advantage of culture techniques is the distinct proof of residence in the sampled anatomical site by documentation of ‘viable bacteria’ from biopsy samples taken from a specific segment of the gut. The culture yield from these samples represents the ‘live’ mucosa-associated bacterial population of that segment, while the best sequencing methods cannot distinguish viable and non-viable ‘dead’ bacteria. Indeed, enhanced culture techniques coupled with identification of bacterial species using 16S rRNA gene amplification have created the paradigm shift in the field of microbial culturomics [22].

Most of the colonic bacterial species are strict anaerobes and there is a paucity of data that combines 16S rRNA gene assessment of faecal and mucosal samples collected and assessed under anaerobic conditions. Multiple studies have shown that the colonic microbiota is significantly altered after bowel preparation prior to colonoscopy, [23–26] while we have identified only one study suggesting that bowel preparation does not affect the composition of the microbiota for more than a month [27]. Previous studies have also documented that personalised dietary changes can alter the gut microbiota within an individual. These changes can occur quite rapidly with change in dietary patterns leading to dramatic shifts in the gut bacterial population [28–30]. This has relevance when considering the dietary restrictions placed before colonoscopy wherein subjects are told to avoid dietary fibre for 24 to 48 h prior to the procedure, which may produce an inherent and unseen bias in the determination of the ‘normal’ gut microbiome of that individual. Additionally, any large time gap between the collection of the faecal and biopsy samples may yield results that are not directly comparable as they represent two different time points in the dietary calendar of the subject.

This current study on healthy volunteers is based on paired faecal and biopsy samples obtained from the rectum during flexible sigmoidoscopy (without prior bowel preparation) that were collected on the same day and transported and cultured in an anaerobic environment. This assessment removes the potential confounders affecting previous studies and provides accurate profiling of the anaerobic bacterial species residing loosely adherent to the mucosa and in the intestinal lumen of ‘healthy’ individuals. The study also underscores the importance of stringent anaerobic culture techniques in the assessment of the gut microbiota in addition to the 16S rRNA gene sequencing in this healthy cohort.

## Methods

### Study subjects

Ten healthy subjects aged 27–43 years with normal BMI (defined as 18–25), and no history of any chronic diseases were recruited to the study. Details of exclusion criteria and individual biometric information can be found in Table 1, and Additional file 1: Table S1. Dietary intake was assessed using a self-administered Scottish Collaborative Group food frequency questionnaire (SCG FFQ, version 6.6) which is a validated, semi‐quantitative dietary assessment instrument [31]. The SCG FFQ covers 169 food items grouped into 21 categories (e.g., breads and breakfast cereals) and was used to describe each participant’s habitual diet over the previous 3 months. All subjects provided signed informed consent before participation. The study was performed in accordance with the principles of the Declaration of Helsinki and the study protocol was approved by Rowett Human Studies Ethical Review Panel and the Ethics Committee of North of Scotland Research Ethics Service (Reference 17/NS/0112).

**Table 1:** Volunteer demographics

Volunteer	Age (years)	Gender	Body mass index (kg/m^2^)	Smoke	Antibiotic (last 6 months)	Bowel movement
P1	40	Female	21.7	No	No	More than once a day
P2	30	Female	23.2	No	No	More than once a day
P3	43	Female	24	No	No	Once/day
P4	33	Female	29	No	No	Once/day
P5	28	Male	21.2	No	No	Once/day
P6	29	Female	21.8	No	No	2–3 times/week
P7	28	Female	23.8	No	No	Once/day
P8	36	Female	18.7	No	No	Once/day
P9	37	Female	24	No	No	2–3 times/week
P10	27	Female	24.5	No	No	Once/day

Exclusion criteria included history of CVD, diabetes, bowel disease, autoimmune disorders, cancer and mental health issues; other chronic diseases; regular probiotic consumption (none in previous 2 weeks); antibiotic therapy within previous 6 months; other prescribed medications (including anticoagulants)

### Faecal and biopsy sample collection and processing

**Fig. 1:**
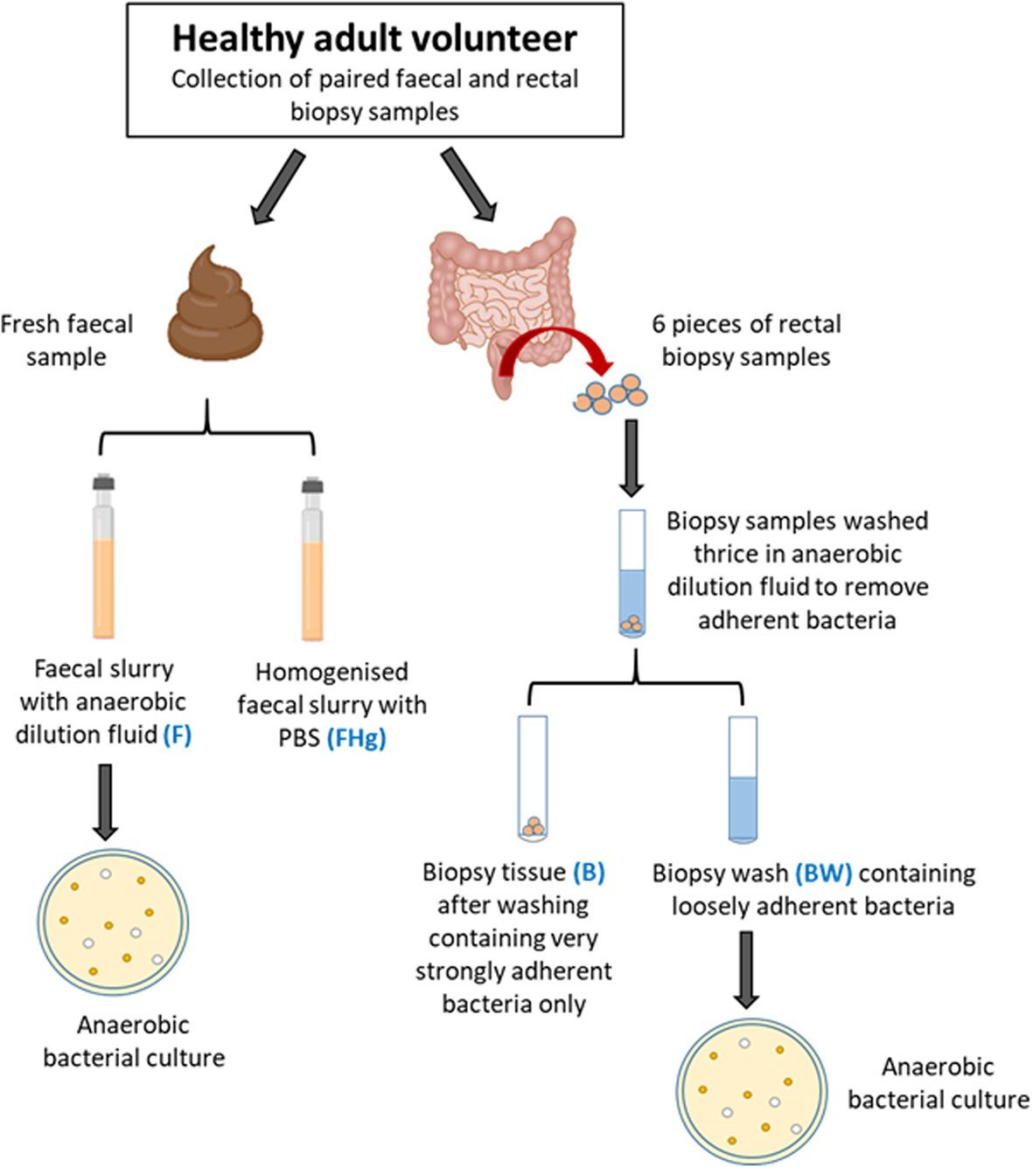
Schematic diagram outlining sample collection and processing. Four different sample types were analysed**:** faecal (F), faecal homogenised (FHg), biopsy tissue (B) and biopsy wash (BW) samples. DNA was extracted from all 4 sample types for microbial community profiling using Illumina sequencing

Enrolled volunteers were invited to the Albyn Hospital, Aberdeen, Scotland where they underwent flexible sigmoidoscopy for collection of rectal biopsy samples. The volunteers did not receive any bowel preparation. Six mucosal pinch biopsy samples were collected from the rectum (approximately 10 cm above the anal verge) of each volunteer anaerobically (bowel inflated with CO_2_ during the procedure) and placed immediately into anaerobic tissue transport medium (Cary Blair, Oxoid, UK) to preserve the viability of anaerobic bacteria. Samples were transported to the laboratory immediately in cool bags and were processed within 1–2 h of collection under stringent anaerobic conditions. A schematic of sample collection and processing is presented in Fig. 1.

In the laboratory, all six biopsy samples from each donor were transferred into 1 ml of anaerobic dilution fluid along with the surrounding agar transport medium, vortexed, washed three times in anaerobic dilution fluid (0.25% Bacto yeast extract, 0.4% NaHC0_3_, 1% Liquid Gold, 15% Mineral 1 and 2 solution, 0.1% Tween 80, Resazurin and Cysteine HCl) [32] vortexing the mix each time, and the three washes were then pooled together (2 ml). Subsequently, serial dilutions of the pooled wash were cultured as described below (for 6/10 samples). The remaining pooled biopsy wash (BW) was immediately centrifuged at 14,500 × *g* for 10 min, the resultant pellet was re-suspended in a total volume of 900 µl of PBS solution with 30% glycerol and then split into two aliquots of 450 µl. One aliquot was stored at − 70 °C and the other was processed immediately for DNA extraction as described below. The remaining biopsy tissues (B) were snap frozen in liquid nitrogen and stored at – 70 °C for later DNA extraction for assessment of microbiota composition.

Fresh paired faecal samples were obtained at the same time from volunteers and were transported to the laboratory in cool bags containing frozen icepacks and processed within 6 h of defecation. These faecal samples were processed in two parts. Firstly, an aliquot of sample was scooped from the unexposed inner part of the faecal material (to ensure viable anaerobic bacteria were sampled), suspended in anaerobic dilution fluid (1 g faeces per 9 ml buffer–10^−1^ dilution) aseptically whilst flushing the tube with CO_2_ following the anaerobic Hungate method [33]. This vortexed faecal suspension (F) was then serially diluted and plated directly onto selective media as described in the next Sect. (6/10 samples). The remainder of the first dilution (10^−1^) was centrifuged at 14,500 × *g* for 10 min, the resultant pellet was re-suspended in a total volume of 900 µl of PBS solution with 30% glycerol and then split into two aliquots of 450 µl. One aliquot was stored at − 70 °C and the other was processed immediately for DNA extraction. Secondly, a further aliquot of 5 g, hand-homogenised, faecal sample was suspended in 10 ml PBS solution with 30% glycerol and processed using the GentleMACS ™ Dissociator (Miltenyi Biotec Ltd., UK) to create a homogenised faecal sample (FHg). DNA was extracted immediately from a 450-µl aliquot of the FHg sample and the rest was stored at − 70 ºC (Fig. 1).

### Bacterial anaerobic culture, isolation and purification of strains

All samples were processed and cultured under strict anaerobic conditions in a Whitley MACS MG-1000 anaerobic workstation (gas composition was 10% carbon dioxide, 10% hydrogen, 80% nitrogen) at 37 °C. The pooled biopsy wash samples (BW) were serially diluted (tenfold dilution) in anaerobic dilution fluid from 10^−1^ to 10^−5^ and 100 µl of four dilutions (10^−2^, 10^−3^, 10^−4^ and 10^−5^) were plated on four different media to maximise the likelihood of culturing diverse taxa. For the faecal sample (F), the first tenfold dilution was vortexed for 3 min to mix thoroughly and subsequently diluted by tenfold serial dilutions through to 10^−9^ dilutions. 100 µl of four highest dilutions (10^−6^, 10^−7^, 10^−8^ and 10^−9^) were then plated onto the four different media plates. The media used for isolation from F and BW samples were yeast extract-casein hydrolysate-fatty acids (YCFA) agar [34] supplemented with 1% autoclaved human faecal fermentor waste [35] and one of four different carbohydrate substrate combinations; (1) glucose, soluble potato starch and cellobiose (GSC, Sigma), (2) commercial mucin from porcine stomach (Type III, Sigma) (3) fructans; vivinal galactooligosaccharide (GOS, Friesland Campina Domo, the Netherlands) and Synergy1 (gifted by BENEO-Orafti, Belgium) or (4) potato starch (Sigma) and β-glucan (Polycell Technologies Glucagel) at 0.2% (w/v) of each substrate. The agar plates were incubated for 48 h anaerobically until colonies were observed. Distinct single colonies representing all the various colony morphologies present were picked from the plates and sub-cultured onto the same respective selective plates to re-purify the isolates. A second round of purification was carried out by sub-culturing these re-purified colonies to ensure that pure single strains were isolated. Concurrently, replica plates of all isolates were incubated aerobically at 37 °C for 24 h. All strictly anaerobic, purified isolates were finally inoculated in M2GSC broth [36] in 7.5 ml aliquots in Hungate tubes, sealed with butyl rubber septa (Bellco Glass) and grown anaerobically at 37 °C for 24 h. Purity of broth cultures was checked by Gram staining and the purified isolates stored as glycerol stocks at − 70 °C.

### Bacterial identification by 16S rRNA gene sequencing

Cell pellets obtained from 1 ml of culture were resuspended in 50 μl of sterile distilled H_2_O and served as templates for direct colony PCR using Ready Mix Red Taq PCR Mix (Sigma, UK). Near full length 16S rRNA gene sequences were amplified with a universal primer set fD1 (5’ AGAGTTTGATCCTGGCTCAG 3’) and rP2 (ACGGCTACCTTGTTACGACTT) [37] and PCR amplifications were performed as described previously [38]. The amplified PCR products were purified with multiscreen micro 96 well plates and vacuum filtered (Millipore) according to the manufacturer’s instructions and directly sequenced with primers fD1, rP2, 519f (CAGCMGCCGCGGTAATWC) and 519r (GWATTACCGCGGCKGCTG) at Eurofins Genomics (Germany). Similarity of the 16S rRNA gene sequences from the isolates to those from other organisms was compared with all sequence data in GenBank, using the BLAST algorithm [39]. Sequences that had a similarity percentage lower than 98.65% were defined as new bacterial species and those less than 95% as new bacterial genera [40]. Phylogenetic analysis was performed using MEGA X software [41], distances were calculated according to Kimura’s two-parameter model and a phylogenetic tree was generated using the maximum likelihood algorithm. After construction, the tree was edited using the Interactive Tree of Life website (iTOL) [42].

### Short-chain fatty acid (SCFA) production

SCFA and other fermentation acid formation was assessed in culture supernatants (1 ml) by gas chromatography as described previously [43]. Briefly, following derivatisation of the samples using N-tert-butyldimethylsilyl-N-methyltrifluoroacetamide (MTBSTFA), the samples were analysed using a Hewlett Packard (Palo Alto, CA, USA) gas chromatograph fitted with a fused silica capillary column using helium as the carrier gas. The SCFA concentrations were calculated from the relative response factor with respect to the internal standard two-ethylbutyrate and external standard (a standard mixture of six SCFAs in distilled water).

### DNA extraction for microbial community profiling

DNA was extracted from the faecal and biopsy samples using the FastDNA Spin kit for soil (MP Biomedicals, UK). For the fresh F, FHg and BW samples, 450 μl were placed in lysing matrix E tubes and 978 μl of sodium phosphate buffer and 122 μl MT buffer were added to each tube, which was processed following the manufacturer’s instructions. DNA was eluted in 100 μl FastPrep elution buffer and quantified by Qubit 2.0 Fluorometer (Life Technologies Ltd., UK). For the biopsy tissue samples (B), an additional digestion step was included prior to extraction of DNA. Proteinase K (30 μl) and tissue lysis buffer (ATL buffer, 180 µl, Qiagen, UK) were added to the washed biopsy pieces (six pieces per volunteer) and incubated for 18 h at 56 °C to ensure complete lysis of the biopsy material [44]. DNA extraction was then carried out using the entire lysed biopsy samples (220 µl approx.) and DNA was eluted in 50 µl FastPrep elution buffer. Purified DNA was stored at – 70 ºC. DNA extracted from all samples (F, FHg, BW and B) were subjected to 16S rRNA gene sequencing to profile the entire bacterial community.

### PCR amplification and amplicon sequencing for microbial community profiling

An amplicon library was generated by PCR amplification of the V1–V2 hypervariable region of bacterial 16S rRNA genes using the barcoded fusion primers MiSeq-27F (5’-AATGATACGGCGACCACCGAGATCTACACTATGGTAATTCCAGMGTTYGATYMTGGCTCAG-3’) and MiSeq-338R (5’-CAAGCAGAAGACGGCATACGAGAT-barcode-AGTCAGTCAGAAGCTGCCTCCCGTAGGAGT-3’), which also contain adaptors for downstream Illumina MiSeq sequencing. Each of the samples was amplified with a unique (12 base) barcoded reverse primer. Initial PCR amplification was undertaken with New England BioLabs Q5 High-fidelity DNA Polymerase, utilizing a per-reaction mix of DNA template (1 μl), 5X Q5 Buffer (5 μl), 10 mM dNTPs (0.5 μl), 10 μM F primer (1.25 μl), 10 μM R primer (1.25 μl), Q5 Taq (0.25 μl) and sterile, deionised water (15.75 μl) to a final volume of 25 μl. PCR cycling conditions were as follows: 2 min at 98 °C; 20 cycles of 30 s at 98 °C, 30 s at 50 °C, 90 s at 72 °C; with a final cycle of 5 min at 72 °C followed by a holding temperature at 10 °C. For BW samples 25 cycles and for B samples 29 cycles were used, due to the lower bacterial load and thus DNA yield. Quadruplicate PCR reactions were set up per DNA sample to ensure adequate yield of amplicons. Following confirmation of adequate and appropriately sized products, the quadruplicate reactions were pooled, and ethanol precipitated. The pooled amplicons were then quantified using a Qubit 2.0 Fluorometer (Life Technologies Ltd., UK) and a sequencing mastermix was created using equimolar concentrations of DNA from each sample which was then cleaned up using AMPure XP magnetic beads (Beckman Coulter, High Wycombe, UK) following manufacturer’s instructions. Negative controls using water instead of DNA samples were extracted alongside the samples following the same protocol, and PCR was amplified using either 20 or 29 cycles and included in the sequencing runs to assess the impact of contamination on the results. Sequencing was carried out on an Illumina MiSeq v3 flowcell producing 300 bp paired end reads. Raw sequencing data has been deposited with the European Nucleotide Archive database under accession number PRJEB35864.

### Bioinformatics and statistical analysis

The quality of the data obtained from Illumina MiSeq sequencing was assessed using FastQC (version 0.11.3) [45] and analysed using the DADA2 software package (version 1.3.1) [46]. The DADA2 pipeline encompasses read filtering and trimming, dereplication, error profiling, sample inference, merging of paired end reads, construction of the sequence table, removal of chimeras and assignment of taxonomy based upon the SILVA database (version 132) [47] both at the genus and the species level. The DADA2 output sequence table was converted to biom format using biomformat software (version 2.1.3) [48], and this data used to assess sequence variant abundances, producing counts for each sample. Diversity analysis was performed using the core_diversity_analyses.py script from QIIME (version 1.9.0) [49], with subsampling set to 13,589 reads per sample. Core diversity analyses calculated five alpha diversity measures (observed species, Chao [50], Shannon Index [51], Simpson Index [52] and Good’s coverage) and two beta diversity measures (Bray–Curtis [53] and Binary Jaccard [54]). Information of taxa numbers at each taxonomic level were also produced. Statistical measurement of sample clustering within PCoA plots was measured using the Adonis statistical test, which implements a PERMANOVA. Differential abundance testing of sequence variants between samples was carried out by converting the biom file to a PhyloSeq object [55] and testing differential abundance using linear discriminant analysis effect size (LEfSe) [56], with each sequence variant only considered at the lowest identifiable taxonomic level, and Corncob (version 0.1.0) [57]. This method collapses sequence variants down to set taxonomic levels and uses beta-binomial regression to identify significant differences. Significance was set as a false discovery rate (FDR) < 0.05. Comparisons were made at the taxonomic levels of phylum, family and genus, between the different sample-groups tested. Figures were made using ggplot2 in RStudio. PCoA plots were visualised using Emperor [58].

### Quantitative polymerase chain reaction (qPCR)

Quantitative PCR (qPCR) was performed in duplicate with iTaqTM Universal SYBR® Green Supermix (Bio-Rad) in a total volume of 10 μl amplifying 2 ng of DNA in a CFX384TM Real-time System (Bio-Rad) as described previously (Chung et al. 2016). Samples were amplified with universal 16S rRNA gene primers UniF (GTGSTGCAYGGYYGTCGTCA) and UniR (ACGTCRTCCMCNCCTTCCTC). Standard curves consisted of tenfold dilution series of quantified, amplified bacterial 16S rRNA genes from *Ruminococcus bromii* L2-63 strain. Relative bacterial concentrations in each sample were estimated by comparing the gene copy numbers calculated using the standard curves. As the DNA extracted (especially from the biopsy samples) consists of a mixture of host and bacterial DNA, the presence of both the human GAPDH gene and bacterial 16S rRNA gene were assessed. The number of GAPDH and bacterial 16S rRNA gene copies in each sample were quantified using validated primer sets with respect to their standard curves, and bacterial DNA content was calculated from the ratio of DNA (ng) encoding 16S rRNA genes and GAPDH genes. Data were analysed using BioRad CFX manager software and the detection limit was determined with negative controls containing only herring sperm DNA.

## Results

Overall, ten healthy subjects with no gastrointestinal symptoms or pre-existing diseases, aged between 27 and 43 years (1 male and 9 females) were recruited to the study. The mean ± standard deviation body mass index of the volunteers was 23.19 ± 2.7 kg/m^2^. Volunteers did not take any antibiotics or prebiotic supplements within the previous 6 months or probiotic supplements including yoghurt in the 2 weeks leading up to the sample donation (Table 1, Additional file 1: Table S1). All volunteers followed their habitual balanced diet with one volunteer consuming no meat or fish (P5) and one volunteer consuming no meat (P8), while four volunteers reported no alcoholic beverages (P3, 5, 8 and 10) (Additional file 2: Table S2).

### 16S rRNA gene sequencing-based microbial profiling

Bacterial DNA extracted from faecal (F and FHg) and rectal biopsy (B and BW) samples was used for 16S rRNA gene sequence analyses. A total of 3,471,838 high quality 16S rRNA gene sequence read pairs were obtained from the different sample types sequenced (B, BW, F, FHg) following quality filtering, equating to 86,796 ± 8599 (mean ± SEM) reads per sample (Additional file 3: Table S3). One sample (P5-BW) had failed to amplify correctly (producing only 117 sequence reads) and was eliminated from further analysis. A total of 4118 different sequence variants and their respective abundances per sample was obtained from the DADA2 analysis. Singleton sequence variants (those present in only a single sample at a single count) were removed to leave 4095 sequence variants. Summary of the sequencing data for each sample type is documented in Additional file 4: Table S4. Rarefaction curves for each of the alpha diversity metrics reached a plateau and a saturation phase, indicating that sample biodiversity was adequately covered with the applied sequencing depth (Additional file 5: Figure S1).

The dominant phyla across all samples were Firmicutes (median relative abundance, 54.06%; interquartile range (IQR) 47.55%, 59.73%), Bacteroidetes (24.34%; IQR 20.29%, 29.17%), Proteobacteria (8.54%; IQR 4.58%, 17.46%) and Actinobacteria (4.66%; IQR 2.26%, 8.07%), together representing > 96% of the total taxa. There were significant inter-individual differences noted in the bacterial profiles of biopsy and faecal samples as highlighted in the PCoA analysis (PERMANOVA; *p* = 0.001) (Additional file 6: Figure S2), with three sample types (F, FHg and BW) from any individual clustering together, but clearly separated from the biopsy samples (Additional file 7: Figure S3).

### Comparison of microbial profiles between the different faecal and rectal biopsy sample types

**Fig. 2:**
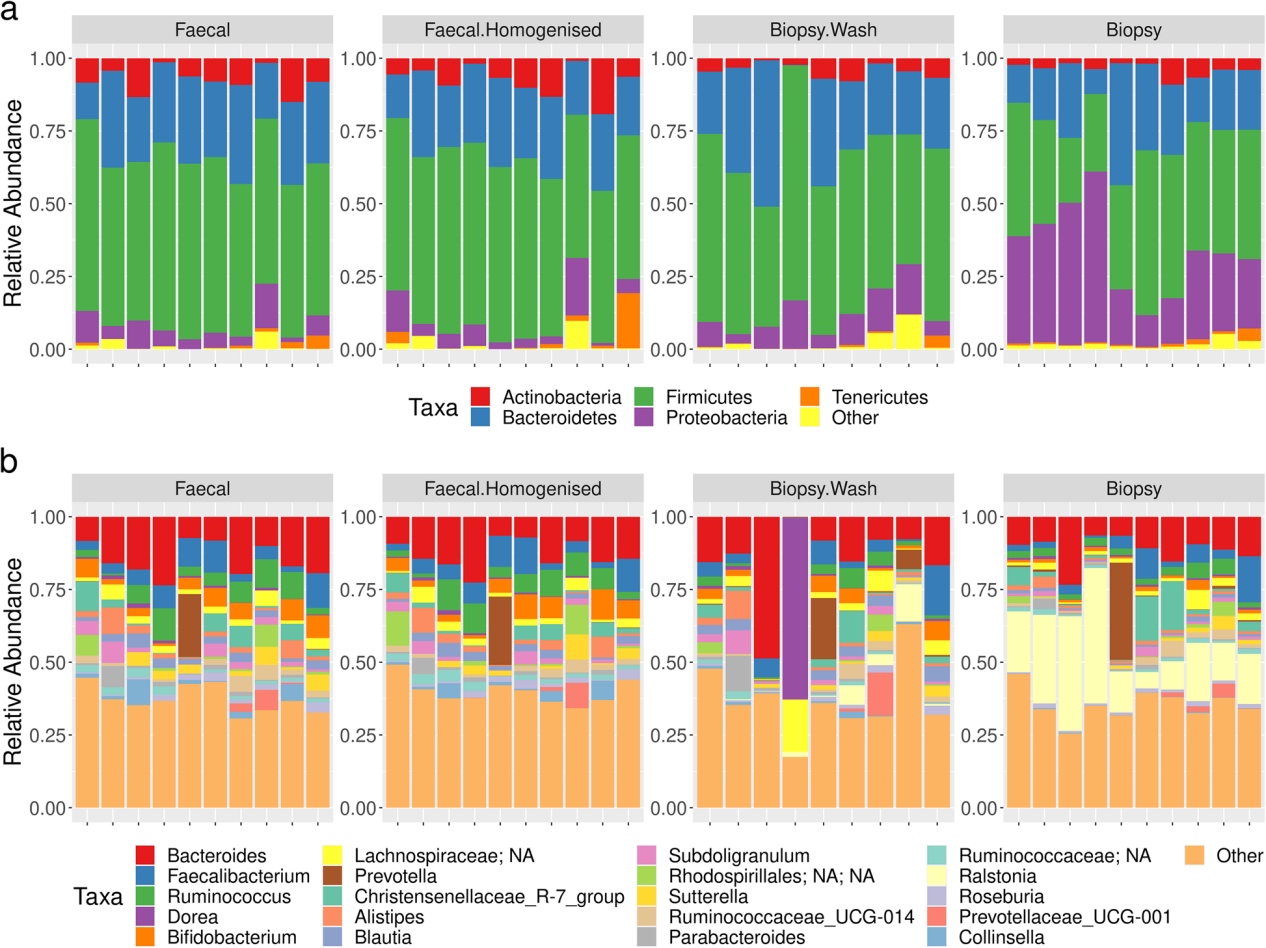
Relative abundance for faecal (F), faecal homogenised (FHg), rectal biopsy tissue (B) and biopsy wash (BW) samples at the (**a**) phylum and (**b**) genus level. The most abundant five phyla and 20 genera are shown

The microbial profiles of the aliquots collected from the whole faecal samples (F) and the faecal homogenised samples (FHg) were compared with each other to decide which of them was best suited for the final comparative analysis. The biopsy washes were also compared to the whole biopsy tissue samples to determine whether there were differences in the composition of loosely adherent and strongly adherent bacteria. Dominant phylum and genus analysis for different sample types indicated that there was considerable similarity between the profiles from the faecal sample, the homogenised faecal sample and the biopsy wash sample from single individuals, with the biopsy tissue samples having a distinctive profile comprising many more Proteobacteria and fewer Firmicutes (Fig. 2, Table 2).

**Table 2:** Dominant phyla detected by amplicon sequence analysis for each sample type. Average median value and interquartile range (IQR) tabulated

	**Faecal**	**Faecal homogenised**	**Biopsy Wash**	**Biopsy**
**Firmicutes**	55.60%	58.30%	55.40%	43.30%
	(IQR 52.9%, 60.3%)	(IQR 52.7%, 61.5%)	(IQR 51.1%, 59.2%)	(IQR 35.7%, 45.5%)
**Bacteroidetes**	27.80%	25.30%	24.30%	20.60%
	(IQR 23.2%, 29.7%)	(IQR 20.3%, 28.0%)	(IQR 21.7%, 36.2%)	(IQR 15.9%, 25.3%)
**Proteobacteria**	5.30%	4.40%	8.50%	28.60%
	(IQR 3.7%, 9.0%)	(IQR 2.8%, 6.7%)	(IQR 5.0%, 14.7%)	(IQR 20.3%, 39.7%)
**Actinobacteria**	8.10%	6.60%	4.50%	3.60%
	(IQR 4.8%, 9.0%)	(IQR 4.5%, 10.0%)	(IQR 2.2%, 6.8%)	(IQR 2.0%, 4.0%)

Differential abundance testing of taxa between the faecal (F) and faecal homogenised (FHg) samples found no significant differences. PCoA plots visualising the results of the Bray–Curtis diversity metric showed tight clustering between F and FHg samples from each individual indicating very close similarity of the microbial profile between these two sample types (Additional file 7: Figure S3).

When comparing biopsy tissue (B) and biopsy wash (BW) samples, there was no such clustering for most volunteers (Additional file 7: Figure S3), and significant differences were found at various taxonomic levels. Four phyla had significantly differential abundance (FDR < 0.05; Additional file 8: Figure S4). Three of these phyla, including Proteobacteria and Acidobacteria, were increased in the B samples relative to the BW samples. Twenty bacterial genera were found to have significant differential abundance (FDR < 0.05; Additional file 9: Figure S5), of which 18 were increased in the biopsy samples. Unsurprisingly, sequences classified as Eukaryota were also more abundant in the biopsy samples. Ten of these more abundant taxa are likely to be contaminants (as discussed below) and were not further investigated. Six of the eight genuinely differentially abundant taxa in biopsy tissue samples were Proteobacteria, consistent with the observed increase in Proteobacteria in biopsy compared to biopsy wash samples at the phylum level. However, the low biomass of the biopsy samples and the associated higher chance of detecting contamination should be kept in mind whilst interpreting these results.

The strong similarity between the bacterial profiles of homogenised (FHg) and non-homogenised (F) faecal samples indicated that they could be represented by a single dataset in the faecal sample: biopsy comparison. There was less similarity between the microbial profile of the B and BW samples. The biopsy washes represent the more abundant, loosely adherent mucosal bacterial population as opposed to the sparse, strongly adherent bacterial subset from the whole rectal biopsy tissue, which includes more species that could be considered as potential pathogens. Thus, the subsequent analysis focussed on comparing the FHg samples and the BW samples as representatives for the best comparison of the faecal bacterial profile and the loosely adherent mucosal bacteria.

### Assessment of contaminant genera in low-biomass biopsy tissue samples

It is important to note that the 20-cycle negative control consisting of ‘DNA’ extracted from the water that had been used to resuspend the DNA produced almost no sequencing reads (133 filtered read pairs), whilst the matched 29-cycle negative control produced 9752 filtered read pairs. Although all other samples yielded more sequencing data, this does indicate the possible amplification of contaminants during the later PCR cycles, a frequent problem in analysis of low biomass samples [59]. These sequencing reads equated to 191 sequence variants that were compared directly with sequence variants identified in the biopsy samples (B and BW, also subjected to extra cycles of PCR) to ensure that only bacteria truly present in the samples and not in the negative control, were focussed on when making conclusions about the analysis. For instance, 10 of the 18 taxa with increased abundance in biopsy samples compared to biopsy wash samples were also present in the 29-cycle negative control (Additional file 9, Figure S5). These were assumed to be contaminants rather than strongly adherent or even invasive bacteria that were unique and truly increased in the biopsy samples. At least three of these 10 taxa have frequently been identified as contaminants in another study [59].

### Similar microbial profile between biopsy wash and faecal homogenised samples

**Fig. 3:**
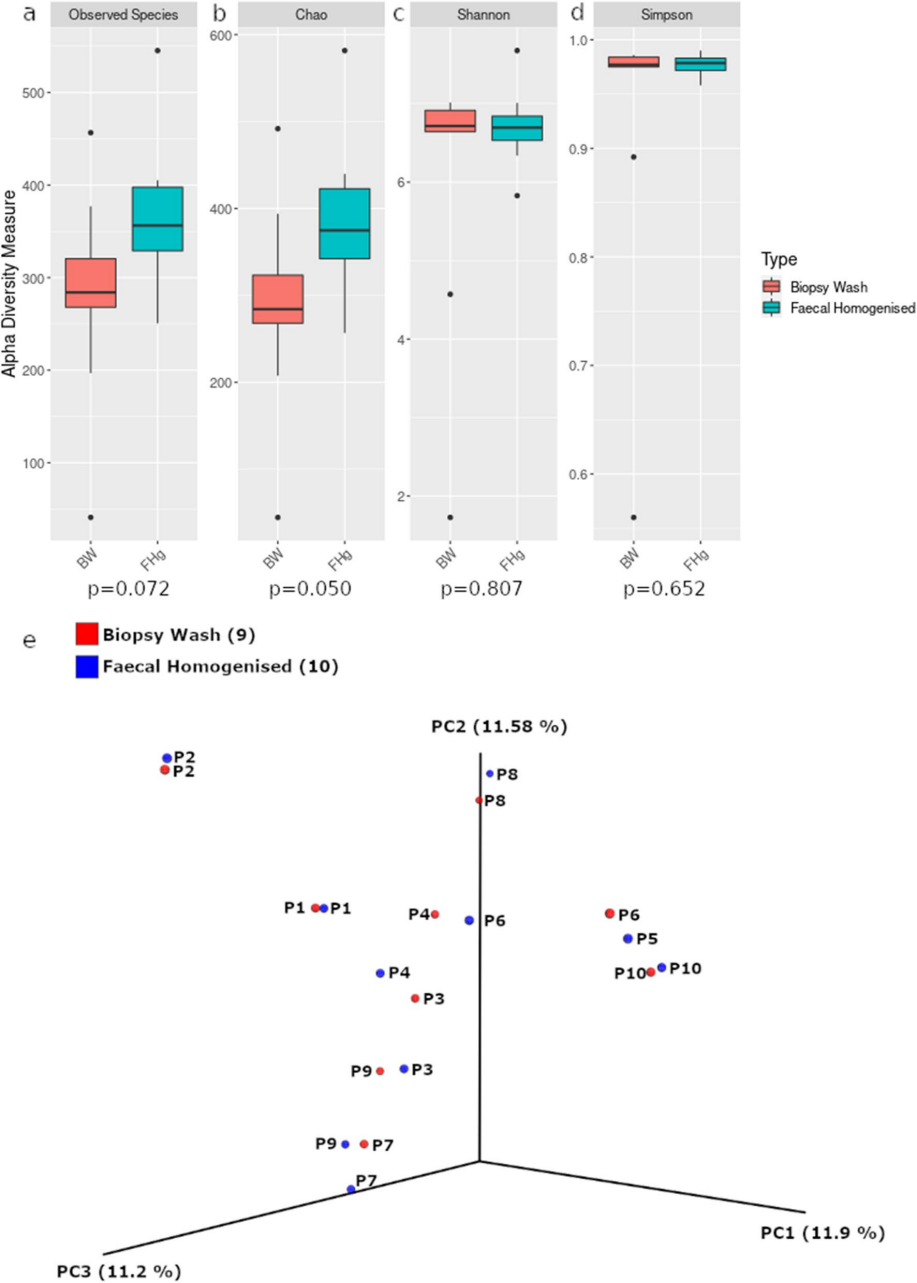
Species diversity comparison between faecal (FHg) and rectal biopsy wash (BW) samples. **a** Observed species, **b** Chao (species richness), **c** Shannon-Weiner diversity index, **d** Simpson diversity index, **e** Beta diversity comparisons, clustering of samples according to sample type by PCoA. Note: Data was not available for the P5-BW sample

**Fig. 4:**
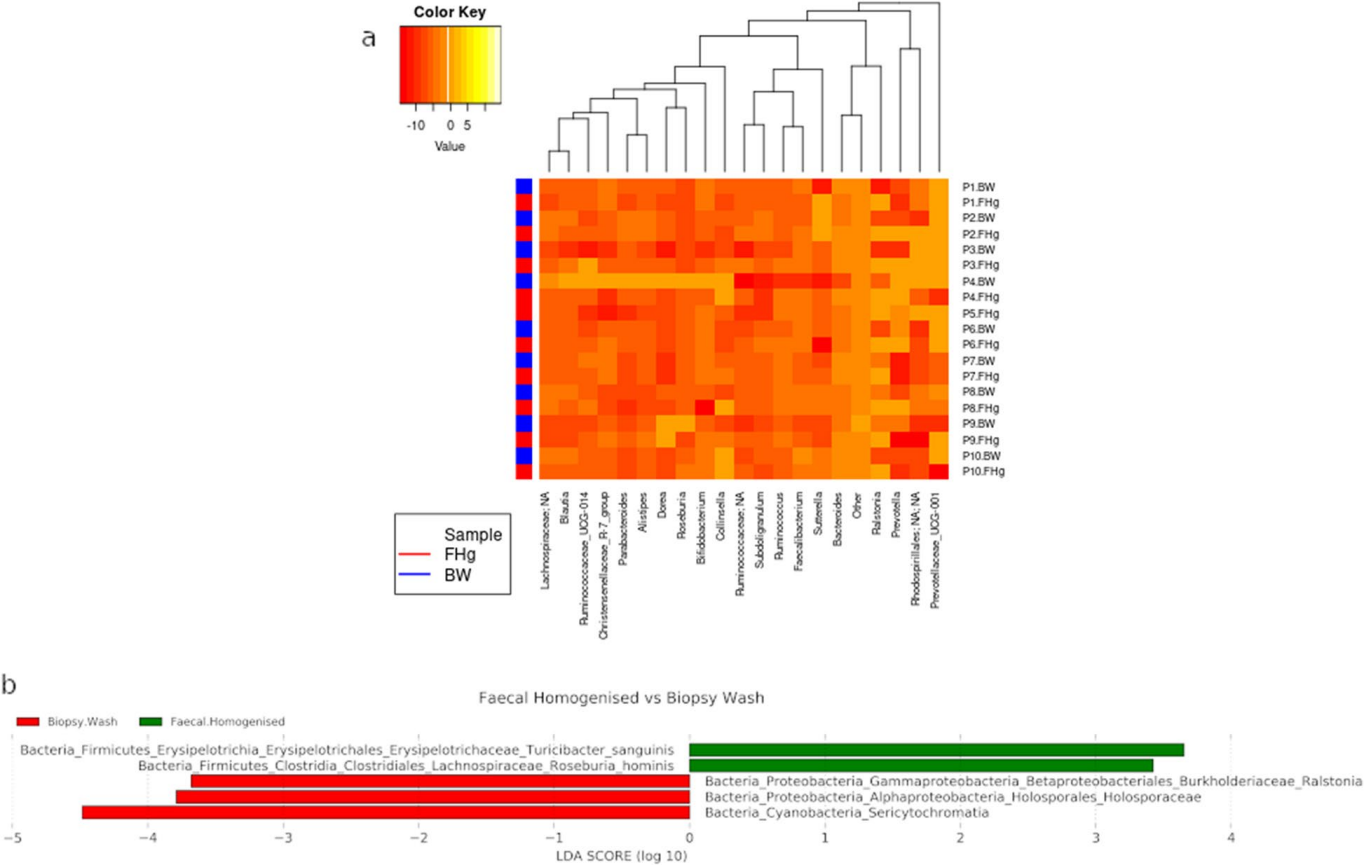
Relative abundance of ASVs in faecal (FHg) and biopsy wash samples (BW) by **a** heat map of Log2count of ASVs. Two sets of colours on the column depicts sample type (red = faecal homogenised, blue = biopsy wash). **b** Significant biomarkers between faecal (FHg) and biopsy wash samples by LEfSe LDA scores

**Fig. 5:**
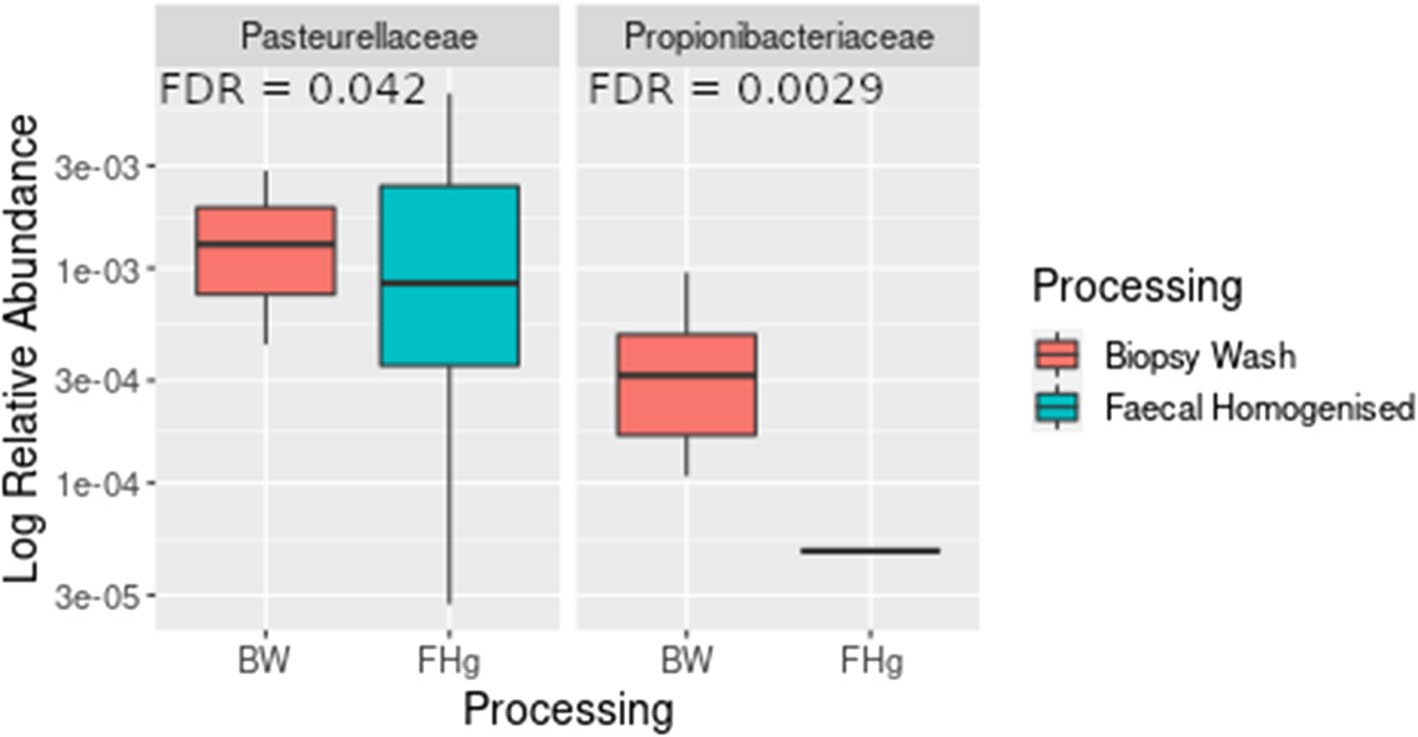
Log relative abundance of taxa with significantly different abundance at the family level between faecal homogenised (blue) and rectal biopsy wash (red) samples

Taxonomic profiling showed limited differences between the faecal homogenised FHg and biopsy wash, (BW) samples at either the phylum (Fig. 2a) or genus (Fig. 2b) level. Bacterial richness and diversity were also comparable between these samples with no significant statistical differences observed between the alpha diversity metrics (Kruskal–Wallis test; observed species *p* = 0.072, Chao *p* = 0.050, Shannon *p* = 0.807, Simpson *p* = 0.652; Fig. 3a–d). Principal coordinates analysis of a Bray–Curtis distance matrix generated from sequence variant tables did not show distinct clustering of biopsy wash and faecal samples (Fig. 3e, PERMANOVA; *p* = 0.972), but showed strong clustering of samples from individual donors. Heatmap of the relative abundances of the most common bacteria classified at the genus level, also showed strong individual similarities between FHg and BW samples (Fig. 4a). Differentially abundant amplicon sequence variants (ASVs) between faecal (FHg) and biopsy wash samples (BW) were analysed by LEfSe to determine which ASVs were driving the differences between sample types. These analyses showed that three ASVs identified as Proteobacteria (*Ralstonia* sp. and *Holosporaceae* sp.) and Cyanobacteria (unclassified Sericytochromatia) were significantly more abundant within the BW samples than the FHg samples. A further two ASVs belonging to the Firmicutes phyla (*Roseburia hominis* and *Turicibacter sanguinis*) were present in significantly higher amounts in the FHg samples compared to BW samples (Fig. 4b). Corncob analysis was also carried out to further investigate the differences in the abundance of ASVs in the two groups which showed that the relative abundance of Propionibacteriaceae and Pasteurellaceae were significantly increased (FDR = 0.0029 and 0.042 respectively) in BW samples compared to FHg samples at the family level (Fig. 5).

### Estimation of total bacterial load by qPCR

Quantitative PCR (qPCR) of DNA extracted from samples was performed in order to estimate the bacterial loads in the respective samples, and to validate the subsequent analyses. The highest bacterial loads (based on 16S rRNA gene copy number) were present in the faecal samples (F and FHg) with very little difference observed between them and were lowest in the biopsy samples (Additional file 10: Figure S6). This was consistent with the results from the bacterial culturing analysis (see below). The low bacterial load and corresponding higher human DNA load in the biopsy samples (BW and B) resulted in a lower proportion of bacterial DNA extracted from these samples and necessitated the additional amplification cycles prior to sequencing as described in the methods section.

### Isolation and culture of anaerobic gut bacteria

For optimal anaerobic cultivation conditions to isolate human gut microbiota, we grew bacteria from faecal (F) and biopsy wash (BW) samples on four different solid media. These media were chosen to represent a wide range of substrates which had previously been shown to have good potential for recovery of the abundant Gram-positive anaerobic gut bacteria (see “Methods” section). A total of 528 anaerobic gut bacteria were cultured as pure isolates from the first six volunteers (BW and F samples). Out of this, 245 bacteria were isolated from BW samples and 283 from F samples. The viable bacterial count from the F samples averaged 10^10^ colony-forming units (CFU)/mL (ranging from 10^9^ to 10^10^ CFU/mL) and the count for the BW samples was lower, averaging 10^5^ CFU/mL (ranging from 10^3^ to 10^6^ CFU/mL). Generally, the F samples contained around 10^5^ more bacteria than the BW samples, with very little difference in the number of colonies growing on the different media (Additional file 11: Table S5). The majority of the 528 isolated bacteria were strictly anaerobic (98%) with only 11 isolates (2%) able to grow aerobically when replated on the isolation media, and all but one of these had initially been cultured from the BW samples. Eight of the ten aerotolerant isolates from BW samples were subsequently identified as Proteobacteria (*Escherichia coli* and *Citrobacter freundii*) and two as Firmicutes (*Staphylococcus epidermidis*). The single aerotolerant isolate cultured directly from the F sample was a facultative anaerobe from the Firmicute phylum (*Enterococcus durans*). The majority of our cultured isolates were obligately anaerobic, confirming that anaerobic conditions had been maintained during collection, transport and growth. Proteobacteria species isolated were not investigated further but were included in the analysis to provide phylogenetic context. Bacterial identification was possible for 498 of 528 isolates (94.32%) (Additional file 12: Table S6).

**Fig. 6:**
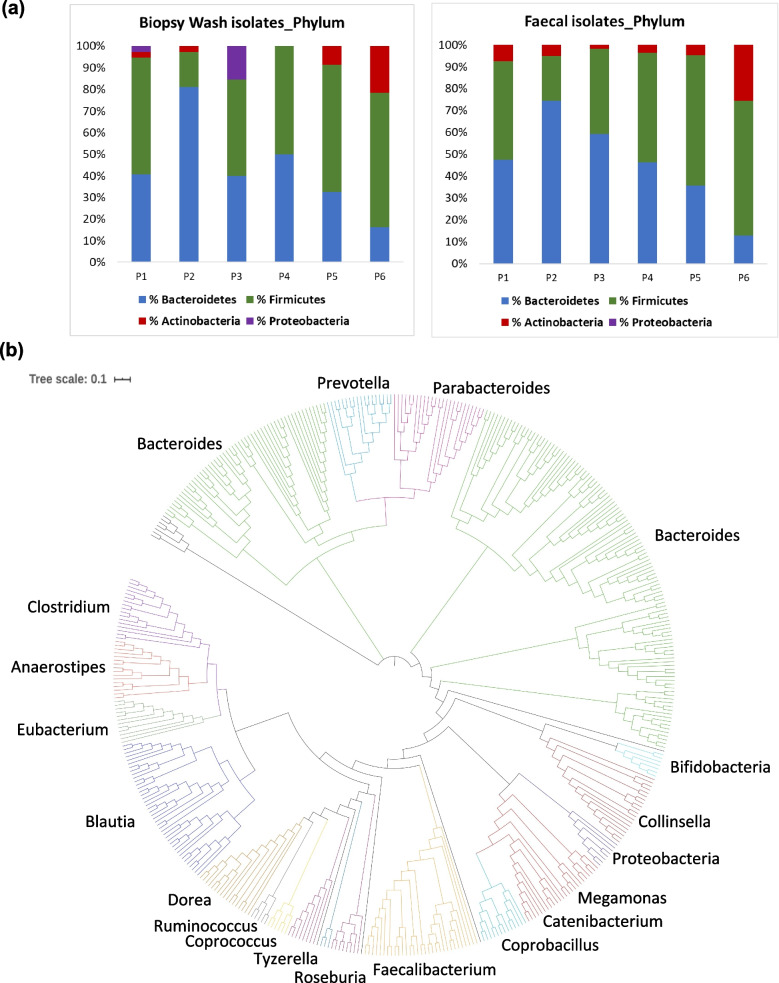
Anaerobic bacterial culture from healthy human faecal (F) and rectal biopsysamples (BW). **a** Phylum distribution of F and BW samples and **b** Phylogenetic tree of bacteria cultured from the six donors constructed from the near full-length 16S rRNA gene sequences. The bar indicates the dissimilarity scale on tree branches. All major genera of bacteria identified are specified

Firmicutes (47%), Bacteroidetes (45%) and Actinobacteria (7%) were the dominant phyla (Fig. 6a) and *Bacteroides*, *Faecalibacterium*, *Prevotella, Eubacterium*, *Collinsella* and *Blautia* species were the dominant genera across all cultured isolates (Fig. 6b, Additional file 12: Table S6). In total, 92 different bacterial species and 22 novel species were identified in this study. The proportions of the three major phyla isolated from F and BW samples were very similar (Correlation coefficient (*r*): Firmicutes 0.899; Bacteroidetes 0.958; Actinobacteria 0.932).

### Comparability between culture and 16S rRNA gene amplicon sequencing results

The taxonomic profile of the cultured anaerobic isolates was very similar to that observed following compositional analysis of extracted DNA by 16S rRNA gene sequencing. In both cases Firmicutes, Bacteroidetes and Actinobacteria were the dominant phyla and *Bacteroides*, *Faecalibacterium*, *Prevotella* and *Eubacterium* species, the dominant genera across all samples. The main difference was the under-representation of Proteobacteria amongst our cultured isolates, presumably because our culture media targeted isolation of obligately anaerobic Gram-positive bacteria, that may thus far be under-represented in culture collections, and was sub-optimal for growth of Proteobacteria.

The taxonomy and relative abundance of identified sequence variants (ASVs) within the faecal, faecal homogenised and biopsy wash samples, were compared to taxonomic assignment and relative abundance of cultured isolates, determined via Sanger sequencing of 16S rRNA gene. This comparison was carried out at both genus and species levels. Of the 92 different cultured isolates collapsed to the level of species, 37 could be matched to ASVs of those species. These taxa accounted for 17% of all ASV counts in the faecal, faecal homogenised and biopsy wash samples. The remaining 55 cultured isolates couldn’t be matched to specific ASVs for two reasons: 28 of the cultured species are not represented in the SILVA species database, and 27 of the cultured isolates were not identified within any samples following 16S rRNA gene sequencing. Of the 37 different cultured isolates collapsed to the level of genus, 32 could be matched to ASVs of those genera. These genera accounted for 51.76% of all ASV counts in the faecal, faecal homogenised and biopsy wash samples. The remaining seven cultured isolates could not be matched to the ASVs as these genera were not present in the SILVA database.

A significant correlation was observed between the abundance of bacteria identified by sequencing (ASVs) and the abundance of cultured isolates (Spearman correlation: genus level–rho = 0.666, *p* = 6.849e − 06, species level–rho = 0.548, *p* = 0.001.

### SCFA and other fermentation acid profiles

We investigated the Firmicutes isolates to assess if there was any difference in metabolite production between the biopsy and faecal isolates. The metabolic activities of these bacterial strains in pure culture were compared by assessing the main acid fermentation products after 24 h growth on rich M2GSC media. SCFA analysis showed that, on the whole, the profiles between biopsy wash isolates and faecal isolates were very similar. The main fermentation product for most isolates was acetate (2–37 mM), followed by lactate (2–27 mM), butyrate (2–25 mM) and succinate (2–14 mM) with many of these bacteria consuming acetate during growth, as previously reported [60] ( Additional file 13: Figure S7). There was no difference between the proportions of butyrate, lactate and acetate produced (Fisher’s two-tailed *P* value, 0.3177, 0.7389 and 0.3262 respectively). Although it appeared that there was more acetate consumption in the BW isolates compared to the faecal isolates, these differences were not statistically significant. The proportion of acetate utilisers was similar between both sample types (Fisher’s two-tailed *p* value, 0.412) but the proportion of butyrate producing, acetate utilising Firmicutes was higher in the faecal samples (Fisher’s two-tailed *p* value, 0.018).

## Discussion

In this study, we compared 16S rRNA gene sequencing-based microbial profiling and culture analysis of paired rectal biopsies and faecal samples, collected from healthy volunteers using strict anaerobic conditions. The deep sequencing analysis confirmed that over 90% of the bacterial species from the biopsy and faecal samples belonged to the four most common bacterial phyla, Firmicutes, Bacteroidetes, Proteobacteria and Actinobacteria, which is consistent with previous published reports [11, 13, 14]. Even though our volunteers were all classed as healthy adults, under 45 years of age and of normal weight, there were significant differences in the bacterial profiles between individuals as noted in the PCoA analysis. Considerable variation has been noted in the gut microbiota of different individuals, frequently driven by varied dietary intake, leading to the suggestion that distinct gut bacterial enterotypes exist [61]. We monitored habitual intake of major food groups across our volunteers and noted considerable differences in the amounts and types of carbohydrates, meat products, and fruits and vegetables consumed by different volunteers. Volunteer 5, the only strict vegetarian with the highest habitual intake of bread and vegetables, also had the highest relative abundance of the genus *Prevotella* in their faecal sample, consistent with previous reports [62, 63]. However, detailed correlation analysis of dietary intakes with individual microbial profiles was not performed due to the small number of participants in this study.

There were no discernible differences following sequence analysis of homogenised faeces samples (FHg) and un-homogenised faecal aliquots (F) confirming the uniformity of bacterial species through the collected samples. This may reduce the need to homogenise faecal samples for analysis in future studies, and validates data obtained by those microbiome testing companies who rely on analysing spot faecal sample cores. However, distinct differences were noted in the bacterial profile of the biopsy wash (BW) and the biopsy tissue samples (B), with a greater relative abundance of Proteobacteria and Acidobacteria noted in the biopsy tissue samples. Quantitative assessment showed significantly reduced bacterial loads from the whole biopsy tissue samples as opposed to the biopsy washes suggesting that the former primarily represents strongly adherent bacterial species that persisted after processing, whereas the BW samples were more representative of the more abundant loosely adherent bacterial population. Such differences have been noted in another human study that showed that colonic lavage samples contained significantly higher numbers of operational taxonomic units (OTUs) compared to corresponding biopsy samples [11]. Our study indicates that the homogenised faecal samples (FHg), un-homogenised faecal aliquots (F) and biopsy washes (BW) have similar bacterial profiles but are distinctly different to the biopsy tissue (B) samples.

Animal biogeographical studies have also shown a preponderance of Proteobacteria in biopsies as opposed to luminal faecal samples obtained from adjacent sections of the bowel [9]. The bacteria residing in the deeper crypts are often distinct from the mucosal bacterial population, with non-fermentative Proteobacteria prevalent [64]. It can be postulated that these bacteria are more difficult to remove from biopsy samples by washing, which could explain why Proteobacteria were more abundant in the biopsy tissue samples in our study.

It has been suggested that laxatives given regularly prior to colonoscopy significantly alter the mucosa-associated gut microbiota with a suggestion that the common laxative, picolax, may also have intrinsic antibacterial effects [24, 26, 65] although other studies have suggested that this may not be the case [27]. Our approach of utilising biopsy washes from samples taken from the rectum without antecedent bowel preparation mitigates these potential biases. Unlike animal models where it is easy to access proximal bowel samples through autopsy, without recourse to laxatives, this is not possible in human subjects. The rectal biopsies obtained from our volunteers were accessed by obtaining mucosal samples avoiding stool pellets, but proximal colonic access by colonoscopy requires prior bowel preparation. However, a study in macaques, found that the microbiota of faecal samples was similar to that of biopsy samples acquired from various segments of the colon during autopsy [9].

Comparison of bacterial profiles of the homogenised faecal samples and the biopsy washes were the key comparison of this study. Significant inter-individual variation was noted in the 16S rRNA gene sequencing profiles from the FHg and BW samples highlighting the uniqueness of the gut microbiota within each individual. However, significant concordance was noted at both the phylum and genus levels between the bacterial profile of FHg samples and the paired BW sample from the same individual. This is contrary to previous observations in healthy volunteers and patients, where the bacterial populations from faeces and colonic biopsies were deemed to be distinct and non-representative of each other [8, 66, 67]. It is worth noting that in these other studies, biopsy samples were taken after bowel preparation which reduces microbial content and therefore affects the microbial profile as previously mentioned. Despite the limitation that our biopsies are rectal rather than colonic, we feel that the remarkable conservation between the bacterial profiles of the paired biopsy wash and faecal samples illustrates that faecal samples are representative of the luminal bacterial community, as well as the biopsy-associated community. The separate analysis of the biopsy wash and biopsy tissue samples hinders an overall comparison of the microbial composition in rectal biopsies with that of corresponding faecal samples.

We propose that the anaerobic transfer of fresh biopsy samples, utilisation of biopsy washes and anaerobic processing of samples used in this study cumulatively led to a representative assessment of the loosely adherent bacterial community. It is well known that most colonic bacteria are strict anaerobes and transport of the small biopsy tissue samples in open contact with oxygen would adversely impact on their survival. This was elegantly demonstrated by the study by Browne et al., where faeces and biopsy samples were placed in anaerobic media within an hour of passage/collection and the entire processing was done in anaerobic surroundings [20]. When sequenced, the faecal samples and the cultured bacterial community shared an average of 93% of raw reads across the six volunteers [20].

Our culture analysis was based on 528 unique bacterial isolates grown from biopsy washes and faecal samples with over 94% of isolates identified to the genus or species level. Only 2% of cultivable bacterial species showed aerobic growth which is consistent with previous reports, and validates our anaerobic methodology [68]. We noted good concordance between the bacterial signatures from anaerobic culture and the microbial profiling based on 16S rRNA gene sequence analysis. There was excellent correlation between bacteria identified from the three major phyla in both methods from faeces and biopsy washes, illustrating that the most abundant faecal bacteria are culturable. This contrasts with a previous older study from three Japanese subjects where a comparative assessment between anaerobic culture and 16S rRNA gene sequencing from faecal material, indicated that a large proportion of the bacteria that could be cultured were uncharacterised [69]. This may be because the comparative genetic databases were not robust enough a decade ago.

The bacterial loads in the different samples were estimated by both viable cell culturing and quantitative PCR. Colony counts from biopsy washes and faecal samples were remarkably similar to the paired estimates from qPCR data. Despite the numerical difference in the bacterial load between both samples, the similarities following microbial profiling strongly suggests that faecal samples reflect biopsy wash samples and can be used as surrogates for assessing loosely adherent mucosa-associated bacteria.

Short chain fatty acids (SCFA) produced by the intestinal microbiota have multiple effects on host physiology and metabolism, most of which are beneficial to health [70, 71]. There were no differences between the proportions of butyrate, acetate and lactate produced by Firmicute bacteria isolated from the faecal and biopsy samples, highlighting that the bacteria isolated from both sites were functionally aligned. In the gut ecosystem, both lactate and acetate are utilised by specialised groups of cross-feeding bacteria, often producing butyrate or propionate [72, 73].

## Conclusions

This study highlights that carefully designed anaerobic conditions facilitate characterization of a representative proportion of human bacteria. We demonstrate that the bacterial profile of faecal samples and rectal biopsy washes is comparable, which will allow researchers to extrapolate results from large gut microbiome studies done on faecal samples to reflect those that occur at the mucosal interface. Remarkable conservation of microbial signatures was noted between paired faecal and rectal biopsy washes from healthy volunteers. However, there were distinct differences noted in the microbial composition of the biopsy tissue samples compared to the other three samples, reflecting the strongly adherent bacterial population. Stringent anaerobic culture is an essential first step in advanced culturomics, which is the technology that will deliver a paradigm leap in the understanding of specific mechanistic interactions between the gut microbiota and individual human hosts.

## Supplementary Information

**Additional file 1:** **Table S1.** List of supplements taken by volunteers.

**Additional file 2:** **Table S2.** Individual energy, nutrient and food intake of all volunteers assessed by food frequency questionnaire (FFQ).

**Additional file 3:** **Table S3.** Read statistics.

**Additional file 4:** **Table S4.** Overview of sequencing data in the different sample groups [F (Faecal), FHg (Faecal homogenised), BW (Biopsy wash) and (B) Biopsy tissue)].

**Additional file 5:** **Figure S1.** Rarefaction plots for every sample for the alpha diversity metrics a) Observed Species, b) Chao, c) Shannon Index, d) Simpson Index and e) Good’s Coverage.

**Additional file 6:** **Figure S2.** PCoA plots based upon Bray Curtis Diversity metrics for different volunteers showing inter-individual differences in the bacterial profile of both biopsy and faecal samples. Each volunteer is represented by a different colour, revealing the four linked samples.

**Additional file 7:** **Figure S3.** PCoA plots based upon Bray Curtis Diversity metrics for different sample types.

**Additional file 8:** **Figure S4.** Log relative abundance of taxa with significantly different abundance at the phylum level between biopsy tissue (red) and biopsy wash (blue) samples.

**Additional file 9:** **Figure S5.** Log relative abundance of taxa with significantly different abundance at the genus level between biopsy tissue (red) and biopsy wash (blue) samples.

**Additional file 10:** **Figure S6.** qPCR estimation of bacterial loads in the different sample types.

**Additional file 11:** **Table S5.** Total viable counts of anaerobic bacteria enumerated on growth plates from biopsy wash (BW) and faecal (F) samples.

**Additional file 12:** **Table S6.** Bacterial identification.

**Additional file 13:** **Figure S7.** Detection of fermentation acids in media following growth of individual isolates in pure culture. Acid production split for biopsy wash (BW) and faecal (F) bacterial isolates. The upward arrow indicates fermentation acid production whilst the downward arrow represents consumption during bacterial growth in pure culture. Bacterial isolate numbers shown on the x-axis correspond to bacterial identities detailed in Additional file 12, Table S6. Data are averages of technical replicates from single cultures.

## Data Availability

The original MiSeq 16S rRNA gene sequence data that support the findings of this study have been deposited in the European Nucleotide Archive database under accession number PRJEB35864.16S rRNA gene sequences derived from the sequencing of PCR products from all cultured bacterial isolates were submitted to GenBank with the accession numbers from MW397642—MW398109 and ON815066-ON815095.

## Abbreviations

ASV: Amplicon sequence variants
B: Biopsy tissue sample
BMI: Body mass index
BW: Biopsy wash sample
CFU: Colony-forming units
DNA: Deoxyribonucleic acid
F: Faecal sample
FDR: False discovery rate
FHg: Homogenised faecal sample
GAPDH: Glyceraldehyde 3-phosphate dehydrogenase
GOS: Galactooligosaccharide
GSC: Glucose, soluble potato starch and cellobiose
IQR: Interquartile range
iTOL: Interactive tree of life
LEfSe: Linear discriminant analysis effect size
MTBSTFA: N-tert-butyldimethylsilyl-N-methyltrifluoroacetamide
OTU: Operational taxonomic unit
PBS: Phosphate-buffered saline
PCR: Polymerase chain reaction
PERMANOVA: Permutational multivariate analysis of variance
qPCR: Quantitative polymerase chain reaction
RESAS: Rural and Environmental Sciences and Analytical Services
rRNA: Ribosomal ribonucleic acid
SCFA: Short-chain fatty acid
SCG FFQ: Scottish Collaborative Group food frequency questionnaire
SEM: Standard error of mean
YCFA: Yeast extract-casein hydrolysate-fatty acids
