# Supplementary material for: Comparison of microbial signatures between paired faecal and rectal biopsy samples from healthy volunteers using next-generation sequencing and culturomics
Source: Microbiome. 2022 Oct 14;10:171. doi: 10.1186/s40168-022-01354-4 (PMC9563177; doi:10.1186/s40168-022-01354-4)
Supplement: Supplementary file 2 — Additional file 1: Table S1. List of supplements taken by volunteers. [file 40168_2022_1354_MOESM1_ESM.docx]

**Additional file 1: Table S1.** List of supplements taken by volunteers.

| **Vol ID** | | **Vitamin Supplement** | | **Dose** | | **Mineral Supplements** | | **Dose** | **Probiotic Supplement*** | | | **Dose** | **Prebiotic supplement** | **Other supplement** | **Dose** |
| --- | --- | --- | --- | --- | --- | --- | --- | --- | --- | --- | --- | --- | --- | --- | --- |
| **P1** | | Berocca multivitamin | | 1 per day | | None | |  | None | | |  | None | None |  |
| **P2** | | Vit D (Boots own) | | 1 per day | | Iron (Boots own) | | 2-3 per week | Yeo valley yoghurt | | | 1 per day | None | None |  |
| **P3** | | None | |  | | None | |  | None | | |  | None | None |  |
| **P4** | | Vit C | | 1 per day | | None | |  | Mueller coffee yoghurt | | | 2-3 per week | None | None |  |
| **P5** | | None | |  | | None | |  | Homemade yoghurt | | | 1 per day | None | None |  |
| **P6** | | Vit D (Sainsbury), multivitamin Supradyn ricarica (Bayer) | | 2-3 per week | | None | |  | Lactoflorene Plus (Montefarmaco OTC) | | | 1 per day | None | None |  |
| **P7** | | None | |  | | None | |  | None | | |  | None | Cod liver oil (Morrisons own) | 1 per day |
| **P8** | | Vit D + Calcium (Osteocare) | | 1 per day | | Vit D + Calcium (Osteocare) | | 1 per day | None | | |  | None | None |  |
| **P9** | | None | |  | | None | |  | None | | |  | None | None |  |
| **P10** | | None | |  | | None | |  | Yoghurt (Lidl) | | | Not specified | None | None |  |
|  |  | |  | |  | |  | | |  |  |  |  |  |  |

*stopped two weeks prior to sample donation if taking regular probiotics.
