# Supplementary material for: Comparison of microbial signatures between paired faecal and rectal biopsy samples from healthy volunteers using next-generation sequencing and culturomics
Source: Microbiome. 2022 Oct 14;10:171. doi: 10.1186/s40168-022-01354-4 (PMC9563177; doi:10.1186/s40168-022-01354-4)
Supplement: Supplementary file 3 — Additional file 2: Table S2. Individual energy, nutrient and food intake of all volunteers assessed by food frequency questionnaire (FFQ). [file 40168_2022_1354_MOESM2_ESM.docx]

**Additional file 2: Table S2.** Individual energy, nutrient and food intake of all volunteers assessed by food frequency questionnaire (FFQ)

| **Energy, nutrient and food intake** | **P1** | **P2** | **P3** | **P4** | **P5** | **P6** | **P7** | **P8** | **P9** | **P10** |
| --- | --- | --- | --- | --- | --- | --- | --- | --- | --- | --- |
| **Total energy (kJ/d)** | 12884 | 7359 | 8200 | 6965 | 30603 | 7695 | 13798 | 7669 | 10710 | 8885 |
| **Carbohydrates (% of energy)** | 50 | 44 | 49 | 47 | 86 | 49 | 43 | 41 | 37 | 29 |
| **Fat (% of energy)** | 31 | 36 | 35 | 34 | 11 | 30 | 36 | 39 | 38 | 46 |
| **Protein (% of energy)** | 17 | 18.25 | 16 | 15 | 4 | 18 | 18 | 20 | 19 | 25 |
| **NSP fibre (% of energy)^a^** | 4 | 7 | 7 | 8 | 2 | 7 | 4 | 7 | 5 | 6 |
| **Foods and food groups** |  | | | | | | | | | |
| **Potatoes (g/d)** | 58 | 28 | 202 | 41 | 0 | 21 | 153 | 0 | 68 | 23 |
| **Pasta, rice, pizza (g/d)** | 356 | 81 | 145 | 153 | 27 | 317 | 282 | 57 | 146 | 71 |
| **Bread (g/d)** | 99 | 7 | 77 | 47 | 115 | 42 | 88 | 0 | 0 | 65 |
| **Breakfast cereals (g/d)** | 63 | 11 | 23 | 46 | 7 | 57 | 87 | 0 | 45 | 24 |
| **Biscuits, pies & cakes (g/d)** | 136 | 52 | 147 | 54 | 0 | 21 | 106 | 0 | 191 | 20 |
| **Milk & dairy products (g/d)** | 295 | 362 | 70 | 210 | 350 | 361 | 685 | 0 | 255 | 121 |
| **Fruits (g/d)** | 262 | 404 | 151 | 68 | 190 | 139 | 223 | 851 | 236 | 184 |
| **Vegetables (g/d)** | 560 | 540 | 249 | 112 | 913 | 483 | 268 | 421 | 194 | 368 |
| **Soups & sauces (g/d)** | 56 | 92 | 3 | 32 | 38 | 100 | 143 | 0 | 64 | 18 |
| **Red & processed meat (g/d)** | 158 | 70 | 92 | 48 | 0 | 4 | 157 | 0 | 239 | 102 |
| **White meat & fish (g/d)** | 77 | 39 | 74 | 40 | 0 | 88 | 91 | 148^b^ | 48 | 261 |
| **Eggs & egg dishes (g/d)** | 6 | 28 | 6 | 41 | 57 | 9 | 21 | 0 | 71 | 46 |
| **Snacks & nuts (g/d)** | 38 | 25 | 14 | 20 | 21 | 9 | 29 | 125 | 1 | 50 |
| **Sugar, chocolate, sweet spreads (g/d)** | 29 | 9 | 0 | 18 | 136 | 1 | 15 | 0 | 7 | 6 |
| **Alcoholic beverages (ml/d)** | 185 | 94 | 0 | 101 | 0 | 176 | 497 | 0 | 232 | 0 |
| **Non-alcoholic beverages (ml/d)** | 1796 | 1161 | 1080 | 901 | 1291 | 1180 | 2037 | 1490 | 1667 | 777 |

^a^ NSP Non-starch polysaccharides, ^b^ Only consumes fish.
