# Supplementary material for: Comparison of microbial signatures between paired faecal and rectal biopsy samples from healthy volunteers using next-generation sequencing and culturomics
Source: Microbiome. 2022 Oct 14;10:171. doi: 10.1186/s40168-022-01354-4 (PMC9563177; doi:10.1186/s40168-022-01354-4)
Supplement: Supplementary file 4 — Additional file 3: Table S3. Read statistics. [file 40168_2022_1354_MOESM3_ESM.docx]

**Additional file 3: Table S3.** Read statistics.

| **Sample** | **Raw Read Pairs** | **Filtered Read Pairs** |
| --- | --- | --- |
| P1-B | 58413 | 34815 |
| P1-BW | 93956 | 59716 |
| P1-F | 191203 | 129727 |
| P1-FHg | 217410 | 151487 |
| P10-B | 68545 | 45800 |
| P10-BW | 60822 | 39108 |
| P10-F | 192338 | 131404 |
| P10-FHg | 257637 | 171563 |
| P2-B | 38316 | 25252 |
| P2-BW | 32708 | 21995 |
| P2-F | 178212 | 118909 |
| P2-FHg | 176355 | 117978 |
| P3-B | 44112 | 28759 |
| P3-BW | 100043 | 66028 |
| P3-F | 125651 | 75281 |
| P3-FHg | 191261 | 130423 |
| P4-B | 41898 | 24595 |
| P4-BW | 98523 | 60867 |
| P4-F | 132783 | 83445 |
| P4-FHg | 104605 | 65212 |
| P5-B | 90961 | 59702 |
| P5-BW | 117 | 86 |
| P5-F | 178230 | 119393 |
| P5-FHg | 195856 | 132344 |
| P6-B | 116206 | 80680 |
| P6-BW | 270983 | 181061 |
| P6-F | 257419 | 163303 |
| P6-FHg | 209747 | 147250 |
| P7-B | 59278 | 39628 |
| P7-BW | 31486 | 20044 |
| P7-F | 120143 | 78485 |
| P7-FHg | 229527 | 151192 |
| P8-B | 32619 | 20364 |
| P8-BW | 48467 | 32907 |
| P8-F | 219593 | 144095 |
| P8-FHg | 225654 | 142164 |
| P9-B | 70597 | 49907 |
| P9-BW | 25103 | 15834 |
| P9-F | 265411 | 184500 |
| P9-FHg | 186481 | 126535 |
| Pos Control 20 strain mix | 168398 | 123319 |
| Pos Control Gill Mix | 89764 | 58840 |
| Neg Control water 29 cycle | 15614 | 9752 |
| Neg Control Water 20 cycle | 637 | 133 |

Biopsy wash (BW), biopsy tissue (B), faecal (F) and faecal homogenised (FHg)

P5-BW, had just 117 reads and probably did not PCR amplify correctly. It was eliminated from further analysis.
