# Supplementary material for: Comparison of microbial signatures between paired faecal and rectal biopsy samples from healthy volunteers using next-generation sequencing and culturomics
Source: Microbiome. 2022 Oct 14;10:171. doi: 10.1186/s40168-022-01354-4 (PMC9563177; doi:10.1186/s40168-022-01354-4)
Supplement: Supplementary file 5 — Additional file 4: Table S4. Overview of sequencing data in the different sample groups [F (Faecal), FHg (Faecal homogenised), BW (Biopsy wash) and (B) Biopsy tissue)]. [file 40168_2022_1354_MOESM4_ESM.docx]

**Additional file 4: Table S4.** Overview of sequencing data in the different sample groups [F (Faecal), FHg (Faecal homogenised), BW (Biopsy wash) and (B) Biopsy tissue)].

|  | **F** | **FHg** | **BW** | **B** |
| --- | --- | --- | --- | --- |
| **Total reads** | 1,228,542 | 1,336,148 | 497,646 | 409,502 |
| **Total ASVs** | 2,180 | 2,296 | 1,642 | 2,108 |
| **Total phyla** | 11 | 9 | 15 | 24 |
| **Total genera** | 176 | 181 | 212 | 318 |

**Note –** The top 6 phyla (in order of abundance: Firmicutes, Bacteroidetes, Proteobacteria, Actinobacteria, Tenericutes, Cyanobacteria) represent 98.3% of the assigned ASVs. Fusobacteria is the next most abundant comprising only 0.32% of sequences.
