# Supplementary material for: Comparison of microbial signatures between paired faecal and rectal biopsy samples from healthy volunteers using next-generation sequencing and culturomics
Source: Microbiome. 2022 Oct 14;10:171. doi: 10.1186/s40168-022-01354-4 (PMC9563177; doi:10.1186/s40168-022-01354-4)
Supplement: Supplementary file 6 — Additional file 5: Figure S1. Rarefaction plots for every sample for the alpha diversity metrics a) Observed Species, b) Chao, c) Shannon Index, d) Simpson Index and e) Good’s Coverage. [file 40168_2022_1354_MOESM5_ESM.docx]

**Additional file 5: Fig. S1.** Rarefaction plots for every sample for the alpha diversity metrics a) Observed Species, b) Chao, c) Shannon Index, d) Simpson Index and e) Good’s Coverage.


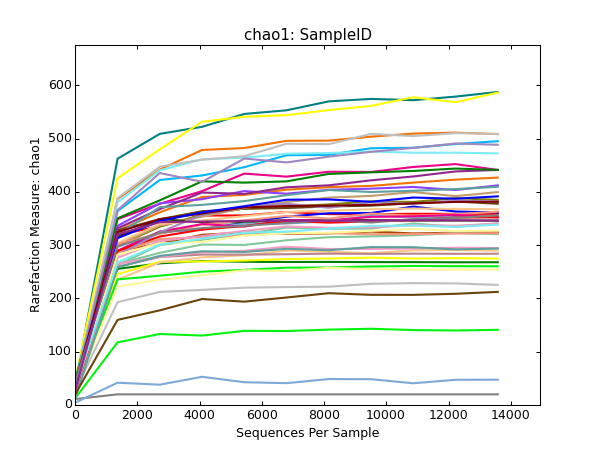

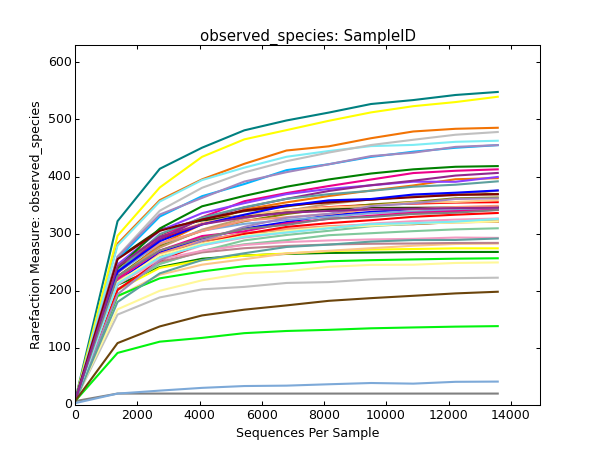


A

B

D

##
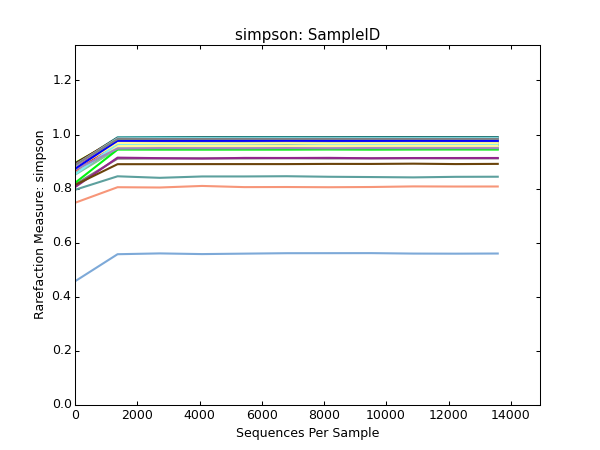

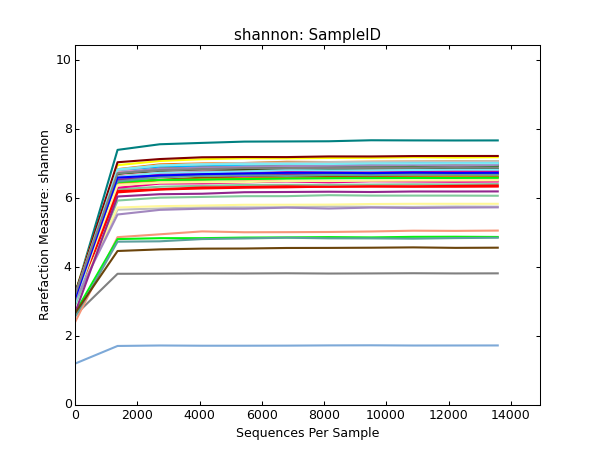


C

##
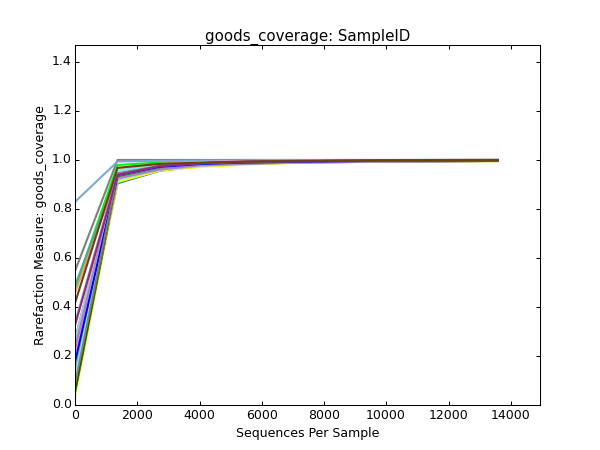


E

**Additional file 5: Table S4. QPCR**
