# Supplementary material for: Comparison of microbial signatures between paired faecal and rectal biopsy samples from healthy volunteers using next-generation sequencing and culturomics
Source: Microbiome. 2022 Oct 14;10:171. doi: 10.1186/s40168-022-01354-4 (PMC9563177; doi:10.1186/s40168-022-01354-4)

**Additional file 6: Fig. S2.** PCoA plots based upon Bray Curtis Diversity metrics for different volunteers showing inter-individual differences in the bacterial profile of both biopsy and faecal samples. Each volunteer is represented by a different colour, revealing the four linked samples.


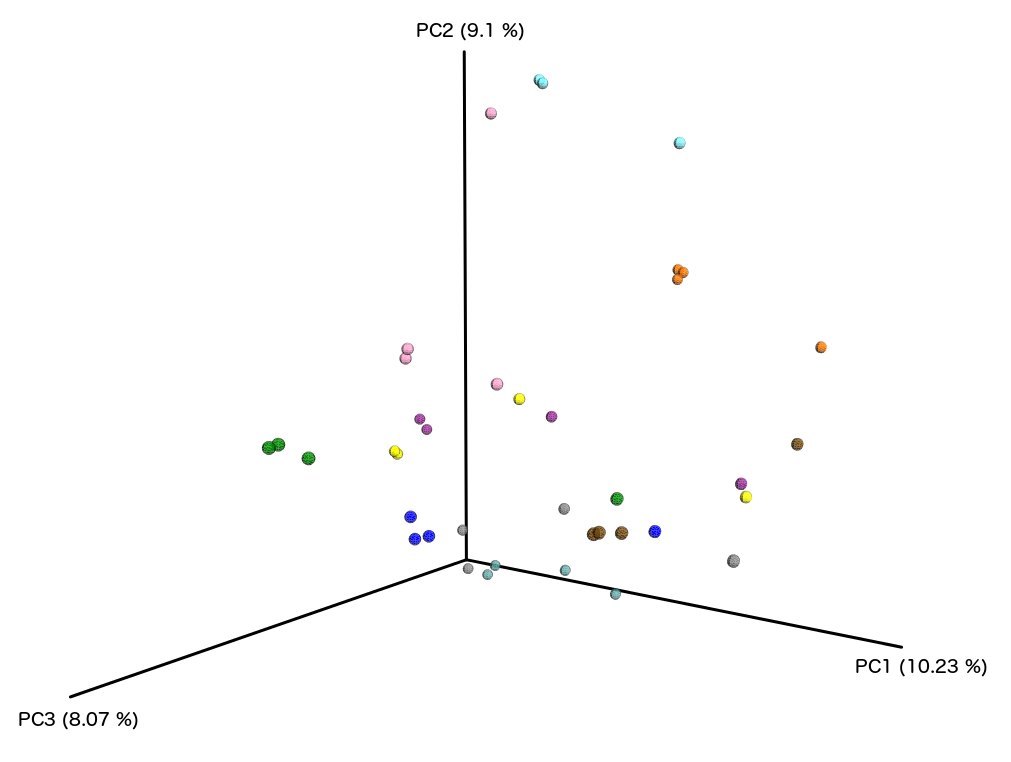

Supplement: Supplementary file 7 — Additional file 6: Figure S2. PCoA plots based upon Bray Curtis Diversity metrics for different volunteers showing inter-individual differences in the bacterial profile of both biopsy and faecal samples. Each volunteer is represented by a different colour, revealing the four linked samples. [file 40168_2022_1354_MOESM6_ESM.docx]
