# Supplementary material for: Comparison of microbial signatures between paired faecal and rectal biopsy samples from healthy volunteers using next-generation sequencing and culturomics
Source: Microbiome. 2022 Oct 14;10:171. doi: 10.1186/s40168-022-01354-4 (PMC9563177; doi:10.1186/s40168-022-01354-4)

**Additional file 7: Fig.S3.** PCoA plots based upon Bray Curtis Diversity metrics for different sample types


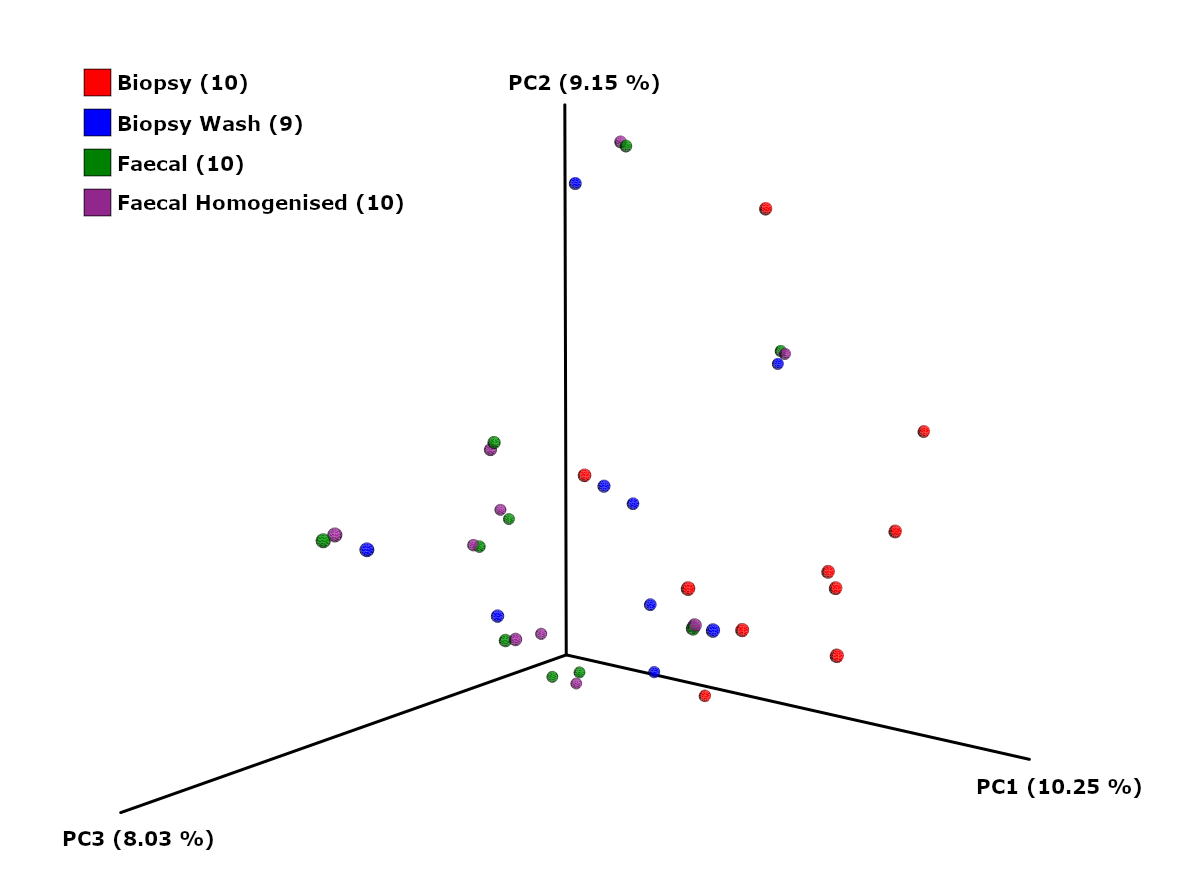

Supplement: Supplementary file 8 — Additional file 7: Figure S3. PCoA plots based upon Bray Curtis Diversity metrics for different sample types. [file 40168_2022_1354_MOESM7_ESM.docx]
