# Supplementary material for: Comparison of microbial signatures between paired faecal and rectal biopsy samples from healthy volunteers using next-generation sequencing and culturomics
Source: Microbiome. 2022 Oct 14;10:171. doi: 10.1186/s40168-022-01354-4 (PMC9563177; doi:10.1186/s40168-022-01354-4)
Supplement: Supplementary file 9 — Additional file 8: Figure S4. Log relative abundance of taxa with significantly different abundance at the phylum level between biopsy tissue (red) and biopsy wash (blue) samples. [file 40168_2022_1354_MOESM8_ESM.docx]

**Additional file 8: Fig.S4.** Log relative abundance of taxa with significantly different abundance at the phylum level between biopsy tissue (red) and biopsy wash (blue) samples.


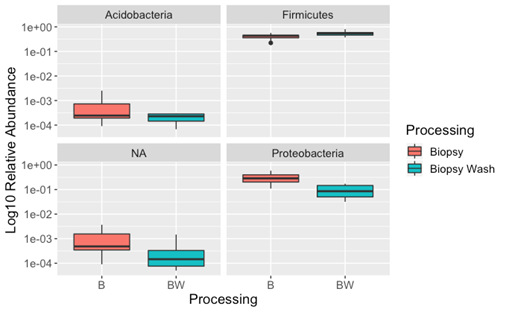


| **Taxa** | **Sample with Higher Abundance** | **False discovery rate (FDR)** |
| --- | --- | --- |
| Bacteria; Firmicutes | Biopsy Wash | 0.026 |
| Bacteria; Proteobacteria | Biopsy | 0.0018 |
| Eukaryota; NA | Biopsy | 0.0018 |
| Bacteria; Acidobacteria | Biopsy | 0.026 |
