# Supplementary material for: Comparison of microbial signatures between paired faecal and rectal biopsy samples from healthy volunteers using next-generation sequencing and culturomics
Source: Microbiome. 2022 Oct 14;10:171. doi: 10.1186/s40168-022-01354-4 (PMC9563177; doi:10.1186/s40168-022-01354-4)
Supplement: Supplementary file 10 — Additional file 9: Figure S5. Log relative abundance of taxa with significantly different abundance at the genus level between biopsy tissue (red) and biopsy wash (blue) samples. [file 40168_2022_1354_MOESM9_ESM.docx]

**Additional file 9: Fig.S5.** Log relative abundance of taxa with significantly different abundance at the genus level between biopsy tissue (red) and biopsy wash (blue) samples.


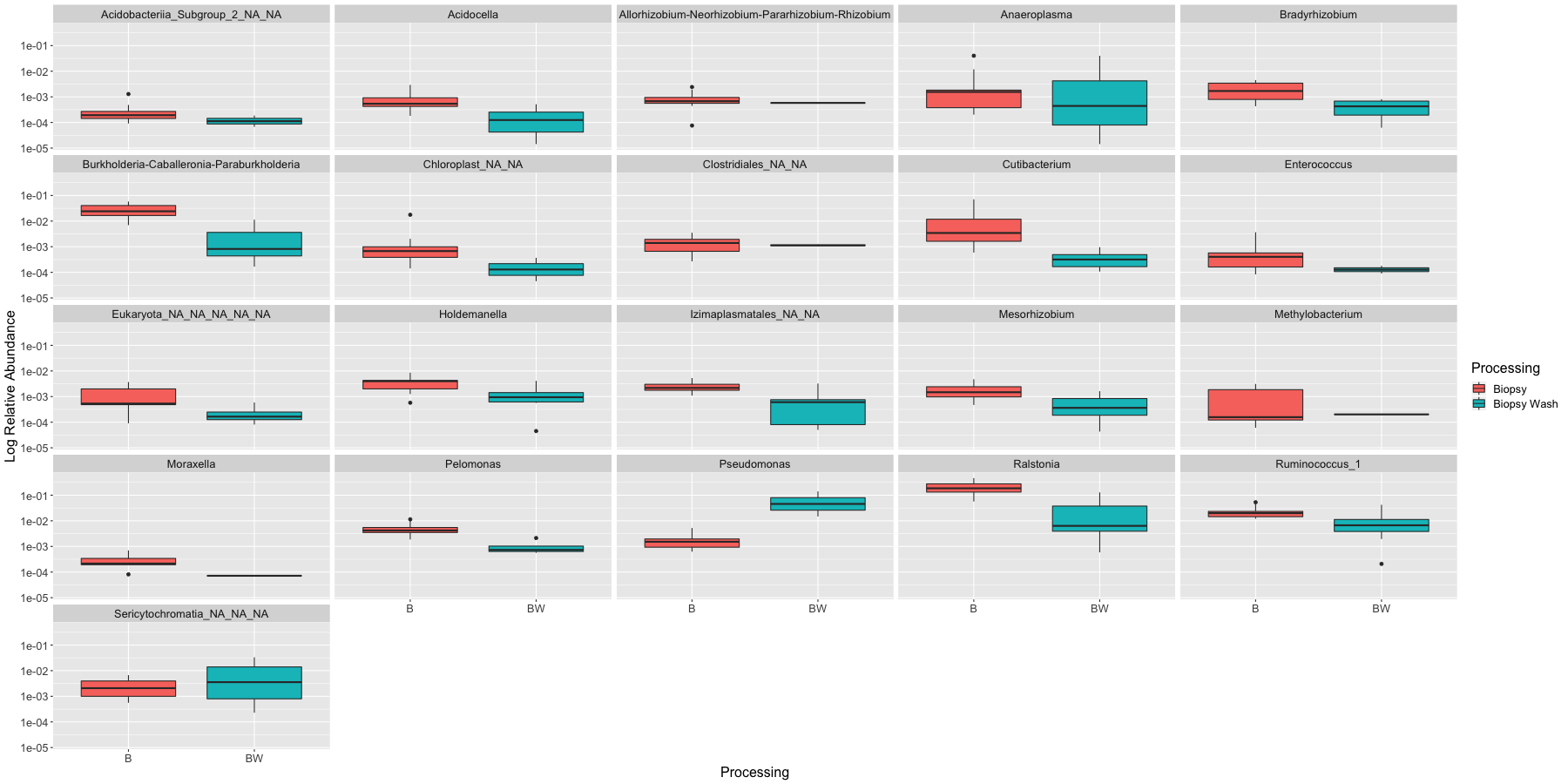


| **Taxa with significantly different abundance at the genus level between biopsy wash and biopsy tissue samples** | **False discovery rate (FDR)** |
| --- | --- |
| **More abundant in biopsy wash** |  |
| Bacteria; Proteobacteria; Gammaproteobacteria; Pseudomonadales; Pseudomonadaceae; Pseudomonas | 0.033 |
| Bacteria; Cyanobacteria; Sericytochromatia; NA; NA; NA | 0.045 |
|  |  |
| **More abundant in biopsy tissue** |  |
| Bacteria; Proteobacteria; Gammaproteobacteria; Betaproteobacteriales; Burkholderiaceae; Pelomonas | 0.000030 |
| Bacteria; Proteobacteria; Gammaproteobacteria; Betaproteobacteriales; Burkholderiaceae; Burkholderia-Caballeronia-Paraburkholderia | 0.00068 |
| Eukaryota; NA; NA; NA; NA; NA [non-bacterial] | 0.0072 |
| Bacteria; Proteobacteria; Alphaproteobacteria; Rhizobiales; Rhizobiaceae; Allorhizobium-Neorhizobium-Pararhizobium-Rhizobium | 0.0092 |
| Bacteria; Proteobacteria; Gammaproteobacteria; Pseudomonadales; Moraxellaceae; Moraxella | 0.020 |
| Bacteria; Firmicutes; Bacilli; Lactobacillales; Enterococcaceae; Enterococcus | 0.020 |
| Bacteria; Proteobacteria; Alphaproteobacteria; Rhizobiales; Rhizobiaceae; Mesorhizobium | 0.039 |
| Bacteria; Acidobacteria; Acidobacteriia; Subgroup_2; NA; NA | 0.040 |
| Bacteria; Proteobacteria; Alphaproteobacteria; Acetobacterales; Acetobacteraceae; Acidocella | 0.049 |
|  |  |
| **Also identified in 29-cycle negative control** (potential contaminants) |  |
| Bacteria; Proteobacteria; Gammaproteobacteria; Betaproteobacteriales; Burkholderiaceae; Ralstonia* | 0.00092 |
| Bacteria; Firmicutes; Clostridia; Clostridiales; NA; NA | 0.0015 |
| Bacteria; Cyanobacteria; Oxyphotobacteria; Chloroplast; NA; NA | 0.0019 |
| Bacteria; Tenericutes; Mollicutes; Izimaplasmatales; NA; NA | 0.0036 |
| Bacteria; Firmicutes; Erysipelotrichia; Erysipelotrichales; Erysipelotrichaceae; Holdemanella | 0.0062 |
| Bacteria; Proteobacteria; Alphaproteobacteria; Rhizobiales; Xanthobacteraceae; Bradyrhizobium* | 0.011 |
| Bacteria; Proteobacteria; Alphaproteobacteria; Rhizobiales; Beijerinckiaceae; Methylobacterium* | 0.020 |
| Bacteria; Tenericutes; Mollicutes; Anaeroplasmatales; Anaeroplasmataceae; Anaeroplasma | 0.033 |
| Bacteria; Firmicutes; Clostridia; Clostridiales; Ruminococcaceae; Ruminococcus_1** | 0.039 |
| Bacteria; Actinobacteria; Actinobacteria; Propionibacteriales; Propionibacteriaceae; Cutibacterium | 0.045 |

* frequently identified as contaminants from kits [59]

** The ASV classed as Ruminococcus_1 was the single most abundant group detected in the negative control
