# Supplementary material for: Comparison of microbial signatures between paired faecal and rectal biopsy samples from healthy volunteers using next-generation sequencing and culturomics
Source: Microbiome. 2022 Oct 14;10:171. doi: 10.1186/s40168-022-01354-4 (PMC9563177; doi:10.1186/s40168-022-01354-4)
Supplement: Supplementary file 11 — Additional file 10: Figure S6. qPCR estimation of bacterial loads in the different sample types. [file 40168_2022_1354_MOESM10_ESM.docx]

**Additional file 10: Fig.S6.** qPCR estimation of bacterial loads in the different sample types.


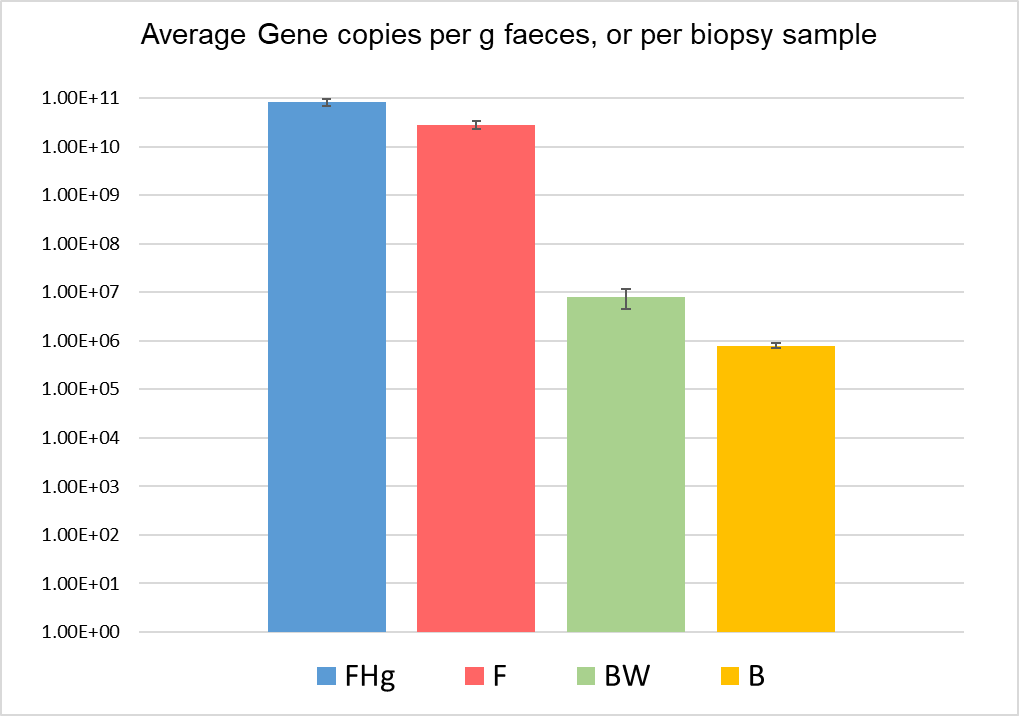


rRNA gene copy number in each sample type (faecal (FHg), faecal (F), biopsy wash (BW) and biopsy (B)) across all 10 volunteers, calculated per g faeces or per pooled biopsy sample.

**
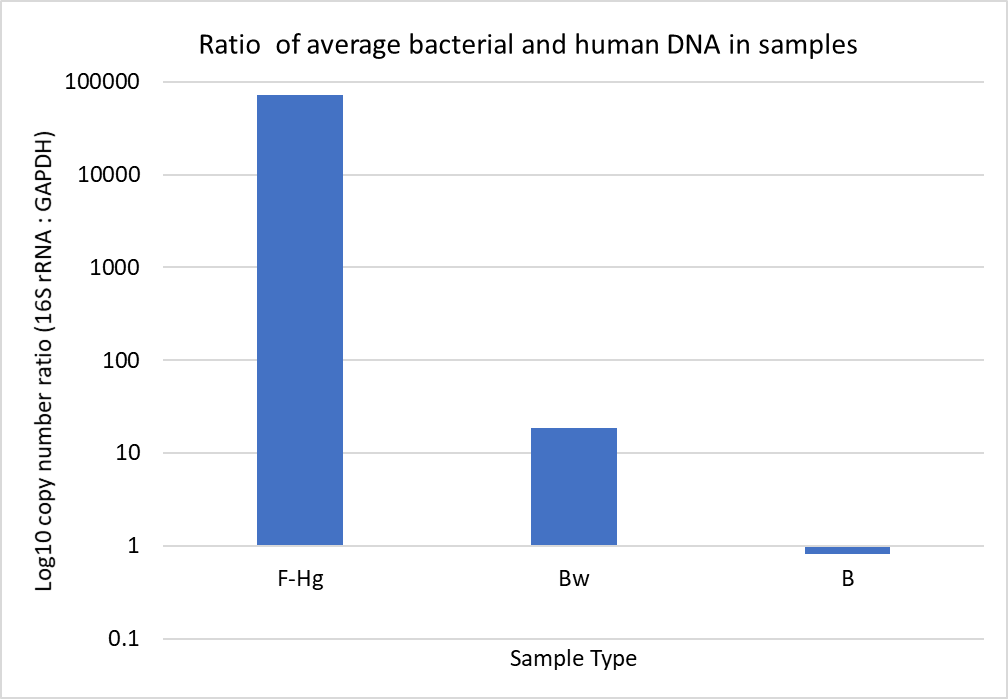
**

Ratio of average bacterial DNA (based on amplification of the 16S rRNA gene) and human DNA (based on amplification of the GAPDH gene) extracted from faecal (FHg), biopsy wash (BW) and biopsy (B) samples from three volunteers (P1, P2 and P3).
