# Supplementary material for: Comparison of microbial signatures between paired faecal and rectal biopsy samples from healthy volunteers using next-generation sequencing and culturomics
Source: Microbiome. 2022 Oct 14;10:171. doi: 10.1186/s40168-022-01354-4 (PMC9563177; doi:10.1186/s40168-022-01354-4)
Supplement: Supplementary file 12 — Additional file 11: Table S5. Total viable counts of anaerobic bacteria enumerated on growth plates from biopsy wash (BW) and faecal (F) samples. [file 40168_2022_1354_MOESM11_ESM.docx]

**Additional file 11: Table S5.** Total viable counts of anaerobic bacteria enumerated on growth plates from biopsy wash (BW) and faecal (F) samples.

Biopsy Wash (BW)

Faeces (F)
