# Supplementary material for: Comparison of microbial signatures between paired faecal and rectal biopsy samples from healthy volunteers using next-generation sequencing and culturomics
Source: Microbiome. 2022 Oct 14;10:171. doi: 10.1186/s40168-022-01354-4 (PMC9563177; doi:10.1186/s40168-022-01354-4)
Supplement: Supplementary file 13 — Additional file 12: Table S6. Bacterial identification. [file 40168_2022_1354_MOESM12_ESM.docx]

Additional file 12: Table S6. Bacterial identification

| **Bacterial Isolate ID** | **Sample type** | **Aerotolerance** | **Closest 16S rRNA BLAST seq match to named bacterium** | **% Identity** | **Phylum** |
| --- | --- | --- | --- | --- | --- |
| PC01 | Biopsy | -ve | Bacteroides fragilis strain ATCC 25285 | 99 | **Bacteroidetes** |
| PC02 | Biopsy | -ve | Catenibacterium mitsuokai strain JCM 10606 | 99 | **Firmicutes** |
| PC03 | Biopsy | -ve | Faecalibacterium prausnitzii strain CNCM_I_4543 | 99 | **Firmicutes** |
| PC04 | Biopsy | -ve | Bacteroides uniformis strain ZL1 | 99 | **Bacteroidetes** |
| PC05 | Biopsy | -ve | [Eubacterium] rectale ATCC 33656 | 99 | **Firmicutes** |
| PC07 | Biopsy | -ve | Butyrate-producing bacterium SR1/1 | 98 | **Firmicutes** |
| PC08 | Biopsy | -ve | Butyrate-producing bacterium T2-132 | 98 | **Firmicutes** |
| PC09 | Biopsy | -ve | Butyrate-producing bacterium T2-132 | 99 | **Firmicutes** |
| PC10 | Biopsy | -ve | Faecalibacterium prausnitzii strain Indica | 99 | **Firmicutes** |
| PC11 | Biopsy | -ve | Bacteroides fragilis strain S14 | 100 | **Bacteroidetes** |
| PC12 | Biopsy | -ve | Catenibacterium mitsuokai strain JCM 10606 | 99 | **Firmicutes** |
| PC13 | Biopsy | -ve | Parabacteroides distasonis strain ATCC 8503 | 99 | **Bacteroidetes** |
| PC14 | Biopsy | -ve | Catenibacterium mitsuokai strain JCM 10606 | 99 | **Firmicutes** |
| PC15 | Biopsy | -ve | Blautia luti | 99 | **Firmicutes** |
| PC16 | Biopsy | -ve | Parabacteroides distasonis strain ATCC 8503 | 99 | **Bacteroidetes** |
| PC18 | Biopsy | -ve | Butyrate-producing bacterium GM2/1 | 99 | **Firmicutes** |
| PC19 | Biopsy | -ve | Collinsella aerofaciens strain D7-88 | 100 | **Actinobacteria** |
| PC20 | Biopsy | -ve | Bacteroides salyersiae strain JCM 12988 | 99 | **Bacteroidetes** |
| PC21 | Biopsy | -ve | Bacteroides fragilis strain S14 | 99 | **Bacteroidetes** |
| PC22 | Biopsy | -ve | Bacteroides uniformis strain:NB-13 | 100 | **Bacteroidetes** |
| PC23 | Biopsy | -ve | Coprococcus comes strain ATCC 27758 | 99 | **Firmicutes** |
| PC24 | Biopsy | -ve | Catenibacterium mitsuokai strain JCM 10606 | 99 | **Firmicutes** |
| PC25 | Biopsy | -ve | Eubacterium rectale M104/1 | 100 | **Firmicutes** |
| PC26 | Biopsy | -ve | Blautia sp. Marseille-P2398 | 99 | **Firmicutes** |
| PC27 | Biopsy | -ve | Bacteroides fragilis strain ATCC 25285 | 100 | **Bacteroidetes** |
| PC28 | Biopsy | -ve | Bacteroides stercoris strain JCM 9496 | 99 | **Bacteroidetes** |
| PC29 | Biopsy | -ve | Bacteroides fragilis isolate 93N_17428 | 100 | **Bacteroidetes** |
| PC30 | Biopsy | -ve | Bacteroides sp. R2F3-5-1 | 99 | **Bacteroidetes** |
| PC31 | Biopsy | -ve | Rumen bacterium 2/9293-18 | 96 | **Firmicutes** |
| PC32 | Biopsy | +++ ve | Staphylococcus epidermidis strain ISLP09 | 100 | **Firmicutes** |
| PC33 | Biopsy | -ve | Ruminococcus lactaris strain ATCC 29176 | 98 | **Firmicutes** |
| PC35 | Biopsy | -ve | Bacteroides fragilis strain S14 | 100 | **Bacteroidetes** |
| PC36 | Biopsy | + ve | Escherichia coli strain BH100 | 99 | **Proteobacteria** |
| PC37 | Biopsy | -ve | Bacteroides intestinalis | 99 | **Bacteroidetes** |
| PC38 | Biopsy | -ve | Parabacteroides distasonis strain ATCC 8503 | 99 | **Bacteroidetes** |
| PC39 | Biopsy | -ve | Catenibacterium mitsuokai strain JCM 10606 | 99 | **Firmicutes** |
| PC40 | Biopsy | -ve | Blautia wexlerae strain AUH-JLD17 | 99 | **Firmicutes** |
| PC41 | Faecal | -ve | Bacteroides uniformis strain ZL1 | 99 | **Bacteroidetes** |
| PC42 | Faecal | -ve | Catenibacterium mitsuokai strain JCM 10606 | 99 | **Firmicutes** |
| PC43 | Faecal | -ve | Faecalibacterium prausnitzii isolate S10H3 | 99 | **Firmicutes** |
| PC44 | Faecal | -ve | Bacteroides salyersiae strain JCM 12988 | 99 | **Bacteroidetes** |
| PC45 | Faecal | -ve | Clostridiaceae bacterium DJF_LS40 | 99 | **Firmicutes** |
| PC46 | Faecal | -ve | Catenibacterium mitsuokai strain JCM 10606 | 99 | **Firmicutes** |
| PC47 | Faecal | -ve | Bacteroides uniformis strain ZL1 | 99 | **Bacteroidetes** |
| PC48 | Faecal | -ve | Faecalibacterium prausnitzii strain CNCM_I_4543 | 99 | **Firmicutes** |
| PC49 | Faecal | -ve | Bacteroides salyersiae strain JCM 12988 | 99 | **Bacteroidetes** |
| PC50 | Faecal | -ve | Blautia wexlerae | 99 | **Firmicutes** |
| PC51 | Faecal | -ve | Parabacteroides distasonis strain ATCC 8503 | 99 | **Bacteroidetes** |
| PC52 | Faecal | -ve | Coprococcus comes strain ATCC 27758 | 99 | **Firmicutes** |
| PC53 | Faecal | -ve | Parabacteroides distasonis strain ATCC 8503 | 99 | **Bacteroidetes** |
| PC54 | Faecal | -ve | Catenibacterium mitsuokai strain JCM 10606 | 99 | **Firmicutes** |
| PC55 | Faecal | -ve | Parabacteroides distasonis strain ATCC 8503 | 99 | **Bacteroidetes** |
| PC56 | Faecal | -ve | Bacteroides uniformis isolate mat-281 | 100 | **Bacteroidetes** |
| PC57 | Faecal | -ve | Collinsella aerofaciens strain D7-88 | 100 | **Actinobacteria** |
| PC58 | Faecal | -ve | Collinsella aerofaciens strain indica | 100 | **Actinobacteria** |
| PC59 | Faecal | -ve | Parabacteroides distasonis | 100 | **Actinobacteria** |
| PC60 | Faecal | -ve | Parabacteroides distasonis strain ATCC 8503 | 99 | **Bacteroidetes** |
| PC61 | Faecal | -ve | Parabacteroides distasonis strain ATCC 8503 | 99 | **Bacteroidetes** |
| PC62 | Faecal | -ve | Bacteroides uniformis strain ZL1 | 99 | **Bacteroidetes** |
| PC63 | Faecal | -ve | Butyrate-producing bacterium L2-50 | 99 | **Firmicutes** |
| PC64 | Faecal | -ve | Catenibacterium mitsuokai strain JCM 10606 | 99 | **Firmicutes** |
| PC65 | Faecal | -ve | Faecalibacterium prausnitzii strain Indica | 99 | **Firmicutes** |
| PC66 | Faecal | -ve | Bacteroides sp. R2F3-5-1 | 99 | **Bacteroidetes** |
| PC67 | Faecal | -ve | Collinsella aerofaciens | 99 | **Bacteroidetes** |
| PC69 | Faecal | -ve | Bacteroides uniformis strain ZL1 | 99 | **Bacteroidetes** |
| PC68 | Faecal | -ve | Lachnospiraceae bacterium G11 | 96 | **Firmicutes** |
| PC70 | Faecal | -ve | Bacteroides uniformis isolate:mat-281 | 100 | **Bacteroidetes** |
| PC71 | Faecal | -ve | Parabacteroides distasonis strain ATCC 8503 | 99 | **Bacteroidetes** |
| PC72 | Faecal | -ve | Ruminococcus faecis strain HBUAS55109 | 99 | **Firmicutes** |
| PC73 | Faecal | -ve | Bacteroides dorei strain HBUAS55078 | 99 | **Bacteroidetes** |
| PC74 | Faecal | -ve | Dorea sp. Marseille-P4003 | 99 | **Firmicutes** |
| PC75 | Faecal | -ve | Catenibacterium mitsuokai strain JCM 10606 | 99 | **Firmicutes** |
| PC76 | Faecal | -ve | Catenibacterium mitsuokai strain JCM 10606 | 99 | **Firmicutes** |
| PC77 | Faecal | -ve | Butyrate-producing bacterium SR1/1 | 99 | **Firmicutes** |
| PC78 | Faecal | -ve | Bacteroides uniformis strain:NB-13 | 99 | **Bacteroidetes** |
| PC79 | Faecal | -ve | Ruminococcus torques L2-14 | 98 | **Firmicutes** |
| PC80 | Faecal | -ve | Bacteroides dorei CL03T12C01, | 100 | **Bacteroidetes** |
| PC81 | Biopsy | -ve | Dorea longicatena strain LCR19 | 100 | **Firmicutes** |
| PC082 | Biopsy | -ve | Parabacteroides distasonis strain SLBE-5 | 100 | **Bacteroidetes** |
| PC083 | Biopsy | -ve | Bacteroides stercorirosoris | 99 | **Bacteroidetes** |
| PC084 | Biopsy | -ve | Bacteroides uniformis strain W19003B | 99 | **Bacteroidetes** |
| PC085 | Biopsy | -ve | Bacteroides stercorirosoris strain JCM 17103 | 99 | **Bacteroidetes** |
| PC086 | Biopsy | -ve | Bacteroides fragilis strain S14 | 99 | **Bacteroidetes** |
| PC087 | Biopsy | -ve | Parabacteroides distasonis JCM 13402 | 99 | **Bacteroidetes** |
| PC088 | Biopsy | -ve | Bacteroides stercorirosoris strain JCM 17103 | 99 | **Bacteroidetes** |
| PC089 | Biopsy | -ve | Bacteroides uniformis strain W19003B | 99 | **Bacteroidetes** |
| PC090 | Biopsy | -ve | Fusicatenibacter saccharivorans HT03-22 | 99 | **Firmicutes** |
| PC091 | Biopsy | -ve | Bacteroides thetaiotaomicron strain SaLBE-19 | 100 | **Bacteroidetes** |
| PC092 | Biopsy | -ve | Parabacteroides distasonis strain SLBE-5 | 100 | **Bacteroidetes** |
| PC093 | Biopsy | -ve | Parabacteroides distasonis strain SaLBE-9 | 99 | **Bacteroidetes** |
| PC094 | Biopsy | -ve | Parabacteroides distasonis strain SaLBE-9 | 99 | **Bacteroidetes** |
| PC095 | Biopsy | -ve | Dorea longicatena strain LCR19 | 100 | **Firmicutes** |
| PC097 | Biopsy | -ve | Anaerostipes hadrus | 100 | **Firmicutes** |
| PC098 | Biopsy | -ve | Dorea longicatena strain JCM 11232 | 100 | **Firmicutes** |
| PC099 | Biopsy | -ve | Bacteroides stercorirosoris strain JCM 17103 | 99 | **Bacteroidetes** |
| PC100 | Biopsy | -ve | Bacteroides fragilis isolate 93N_17428 | 99 | **Bacteroidetes** |
| PC101 | Biopsy | -ve | Parabacteroides distasonis strain SLBE-5 | 100 | **Bacteroidetes** |
| PC102 | Biopsy | -ve | Bacteroides stercorirosoris | 99 | **Bacteroidetes** |
| PC103 | Biopsy | -ve | Bacteroides fragilis strain S14 | 99 | **Bacteroidetes** |
| PC104 | Biopsy | -ve | Bacteroides fragilis isolate 93N_17428 | 99 | **Bacteroidetes** |
| PC106 | Biopsy | -ve | Bacteroides stercorirosoris strain JCM 17103 | 99 | **Bacteroidetes** |
| PC107 | Biopsy | -ve | Bacteroides uniformis strain W19003B | 99 | **Bacteroidetes** |
| PC108 | Biopsy | -ve | Bacteroides thetaiotaomicron | 100 | **Bacteroidetes** |
| PC109 | Biopsy | -ve | Bacteroides uniformis strain W19003B | 100 | **Bacteroidetes** |
| PC110 | Biopsy | -ve | Bacteroides stercorirosoris strain JCM 17103 | 99 | **Bacteroidetes** |
| PC111 | Biopsy | -ve | Bacteroides fragilis strain S14 | 99 | **Bacteroidetes** |
| PC112 | Biopsy | -ve | Bacteroides thetaiotaomicron strain MAME-1 | 99 | **Bacteroidetes** |
| PC113 | Biopsy | -ve | Bacteroides stercorirosoris strain JCM 17103 | 99 | **Bacteroidetes** |
| PC114 | Biopsy | +++ ve | Staphylococcus epidermidis | 100 | **Firmicutes** |
| PC115 | Biopsy | -ve | Bacteroides stercorirosoris strain JCM 17103 | 99 | **Bacteroidetes** |
| PC116 | Biopsy | -ve | Bacteroides thetaiotaomicron strain MAME-1 | 99 | **Bacteroidetes** |
| PC118 | Biopsy | -ve | Parabacteroides distasonis strain SLBE-5 | 100 | **Bacteroidetes** |
| PC119 | Biopsy | -ve | Bacteroides stercorirosoris strain JCM 17103 | 100 | **Bacteroidetes** |
| PC120 | Biopsy | -ve | Bifidobacterium longum strain HBUAS55107 | 100 | **Actinobacteria** |
| PC121 | Faecal | -ve | Bacteroides stercorirosoris strain JCM 17103 | 99 | **Bacteroidetes** |
| PC122 | Faecal | -ve | Lachnospiraceae bacterium | 96 | **Firmicutes** |
| PC123 | Faecal | -ve | Bacteroides nordii strain JCM 12987 | 100 | **Bacteroidetes** |
| PC124 | Faecal | -ve | Butyrate-producing bacterium SS3/4 | 99 | **Firmicutes** |
| PC125 | Faecal | -ve | Bacteroides uniformis strain W19003B | 100 | **Bacteroidetes** |
| PC126 | Faecal | -ve | Blautia wexlerae strain AUH-JLD17 | 99 | **Firmicutes** |
| PC127 | Faecal | -ve | Bacteroides stercorirosoris strain JCM 17103 | 99 | **Bacteroidetes** |
| PC128 | Faecal | -ve | Parabacteroides distasonis strain SaLBE-9 | 99 | **Bacteroidetes** |
| PC130 | Faecal | -ve | Bacteroides nordii strain JCM 12987 | 100 | **Bacteroidetes** |
| PC131 | Faecal | -ve | Parabacteroides distasonis strain SaLBE-9 | 99 | **Bacteroidetes** |
| PC132 | Faecal | -ve | Collinsella aerofaciens strain indica | 99 | **Actinobacteria** |
| PC133 | Faecal | -ve | Bacteroides stercorirosoris strain JCM 17103 | 99 | **Bacteroidetes** |
| PC134 | Faecal | -ve | Parabacteroides distasonis strain JCM 13402 | 99 | **Bacteroidetes** |
| PC135 | Faecal | -ve | Bacteroides stercorirosoris strain JCM 17103 | 99 | **Bacteroidetes** |
| PC136 | Faecal | -ve | Bacteroides stercorirosoris | 99 | **Bacteroidetes** |
| PC137 | Faecal | -ve | Bacteroides stercorirosoris strain JCM 17103 | 99 | **Bacteroidetes** |
| PC138 | Faecal | -ve | Bacteroides stercorirosoris | 99 | **Bacteroidetes** |
| PC139 | Faecal | -ve | Collinsella aerofaciens strain indica | 99 | **Actinobacteria** |
| PC140 | Faecal | -ve | Eubacterium ramulus | 99 | **Firmicutes** |
| PC141 | Faecal | -ve | Parabacteroides distasonis strain SaLBE-18 | 100 | **Bacteroidetes** |
| PC142 | Faecal | -ve | Bacteroides stercorirosoris strain JCM 16102 | 99 | **Bacteroidetes** |
| PC143 | Faecal | -ve | Bacteroides uniformis strain W19003B | 99 | **Bacteroidetes** |
| PC144 | Faecal | -ve | Bacteroides stercorirosoris | 99 | **Bacteroidetes** |
| PC145 | Faecal | -ve | Bacteroides fragilis strain S14 | 99 | **Bacteroidetes** |
| PC146 | Faecal | -ve | Bacteroides fragilis strain S14 | 99 | **Bacteroidetes** |
| PC147 | Faecal | -ve | Bacteroides stercorirosoris strain JCM 17103 | 99 | **Bacteroidetes** |
| PC148 | Faecal | -ve | Parabacteroides distasonis strain SLBE-5 | 100 | **Bacteroidetes** |
| PC149 | Faecal | -ve | Bacteroides uniformis strain W19003B | 99 | **Bacteroidetes** |
| PC150 | Faecal | -ve | Bacteroides stercorirosoris strain JCM 17103 | 99 | **Bacteroidetes** |
| PC151 | Faecal | -ve | Bacteroides thetaiotaomicron strain MAME-1 | 99 | **Bacteroidetes** |
| PC152 | Faecal | -ve | Bacteroides cellulosilyticus strain WH2 | 100 | **Bacteroidetes** |
| PC153 | Faecal | -ve | Bacteroides fragilis isolate 93N_17428 | 99 | **Bacteroidetes** |
| PC154 | Faecal | -ve | Parabacteroides distasonis strain SaLBE-18 | 100 | **Bacteroidetes** |
| PC155 | Faecal | -ve | [Eubacterium] hallii isolate EH1 | 100 | **Firmicutes** |
| PC156 | Faecal | -ve | Bacteroides stercorirosoris strain JCM 17103 | 99 | **Bacteroidetes** |
| PC157 | Faecal | -ve | Bacteroides uniformis strain W19003B | 100 | **Bacteroidetes** |
| PC158 | Faecal | -ve | Lachnospiraceae bacterium | 96 | **Firmicutes** |
| PC159 | Faecal | -ve | Blautia luti strain JCM 17040 | 99 | **Firmicutes** |
| PC160 | Faecal | -ve | Blautia luti strain JCM 17040 | 99 | **Firmicutes** |
| PC161 | Biopsy | -ve | Bacteroides fragilis YCH46 | 100 | **Bacteroidetes** |
| PC162 | Biopsy | -ve | Tyzzerella nexilis Marseille-P3062 | 99 | **Firmicutes** |
| PC163 | Biopsy | -ve | Coprobacillus cateniformis strain ASD2413 | 100 | **Firmicutes** |
| PC164 | Biopsy | +++ ve | Citrobacter freundii complex sp. CFNIH3 | 100 | **Proteobacteria** |
| PC165 | Biopsy | -ve | Bacteroides vulgatus S-18 | 99 | **Bacteroidetes** |
| PC166 | Biopsy | -ve | Tyzzerella sp. Marseille-P3062 | 99 | **Firmicutes** |
| PC167 | Biopsy | -ve | Bacteroides fragilis YCH46 | 100 | **Bacteroidetes** |
| PC168 | Biopsy | -ve | Coprobacillus cateniformis strain ASD2413 | 100 | **Firmicutes** |
| PC169 | Biopsy | -ve | Eisenbergiella tayi strain NML05A014 | 99 | **Firmicutes** |
| PC170 | Biopsy | +++ ve | Citrobacter freundii complex sp. CFNIH3 | 100 | **Proteobacteria** |
| PC171 | Biopsy | +++ ve | Citrobacter freundii complex sp. CFNIH3 | 100 | **Proteobacteria** |
| PC172 | Biopsy | -ve | [Clostridium] symbiosum strain SRB539-5-G-R | 100 | **Firmicutes** |
| PC173 | Biopsy | -ve | Tyzzerella nexilis Marseille-P3062 | 99 | **Firmicutes** |
| PC174 | Biopsy | -ve | Bacteroides fragilis YCH46 | 100 | **Bacteroidetes** |
| PC175 | Biopsy | -ve | Bacteroides fragilis YCH46 | 100 | **Bacteroidetes** |
| PC176 | Biopsy | -ve | Bacteroides fragilis YCH46 | 100 | **Bacteroidetes** |
| PC177 | Biopsy | -ve | Tyzzerella nexilis Marseille-P3062 | 98 | **Firmicutes** |
| PC178 | Biopsy | -ve | Bacteroides fragilis YCH46 | 100 | **Bacteroidetes** |
| PC179 | Biopsy | +++ ve | Citrobacter freundii complex sp. CFNIH3 | 99 | **Proteobacteria** |
| PC180 | Biopsy | -ve | Clostridium symbiosum strain 69 | 99 | **Firmicutes** |
| PC181 | Biopsy | -ve | Tyzzerella nexilis Marseille-P3062 | 99 | **Firmicutes** |
| PC182 | Biopsy | -ve | [Desulfotomaculum] guttoideum strain MB2-A35 | 96 | **Firmicutes** |
| PC183 | Biopsy | -ve | Bacteroides fragilis YCH46 | 100 | **Bacteroidetes** |
| PC184 | Biopsy | -ve | Bacteroides fragilis YCH46 | 100 | **Bacteroidetes** |
| PC185 | Biopsy | -ve | Tyzzerella nexilis Marseille-P3062 | 98 | **Firmicutes** |
| PC186 | Biopsy | -ve | Bacteroides fragilis YCH46 | 99 | **Bacteroidetes** |
| PC187 | Biopsy | +++ ve | Citrobacter freundii complex sp. CFNIH3 | 100 | **Proteobacteria** |
| PC188 | Biopsy | -ve | Coprobacillus cateniformis strain ASD2413 | 100 | **Firmicutes** |
| PC189 | Biopsy | -ve | Bacteroides fragilis YCH46 | 100 | **Bacteroidetes** |
| PC190 | Biopsy | -ve | Bacteroides fragilis YCH46 | 100 | **Bacteroidetes** |
| PC191 | Biopsy | -ve | Bacteroides fragilis YCH46 | 100 | **Bacteroidetes** |
| PC192 | Biopsy | -ve | Tyzzerella nexilis Marseille-P3062 | 99 | **Firmicutes** |
| PC193 | Biopsy | -ve | Tyzzerella nexilis Marseille-P3062 | 98 | **Firmicutes** |
| PC194 | Biopsy | -ve | Bacteroides vulgatus ATCC 8482 | 99 | **Bacteroidetes** |
| PC195 | Biopsy | -ve | Coprobacillus cateniformis strain ASD2413 | 100 | **Firmicutes** |
| PC196 | Biopsy | -ve | Bacteroides vulgatus ATCC 8482 | 99 | **Bacteroidetes** |
| PC197 | Biopsy | +++ ve | Escherichia coli strain KBN10P04869 | 100 | **Proteobacteria** |
| PC198 | Biopsy | -ve | Bacteroides vulgatus strain NMBE-5 | 99 | **Bacteroidetes** |
| PC199 | Biopsy | -ve | Bacteroides fragilis YCH46 | 100 | **Bacteroidetes** |
| PC200 | Biopsy | +++ ve | Escherichia coli strain KBN10P04869 | 100 | **Proteobacteria** |
| PC201 | Faecal | -ve | Bacteroides uniformis | 100 | **Bacteroidetes** |
| PC202 | Faecal | -ve | Bacteroides thetaiotaomicron strain 1203-27010 | 100 | **Bacteroidetes** |
| PC203 | Faecal | -ve | Coprococcus catus GD/7 | 99 | **Firmicutes** |
| PC204 | Faecal | -ve | Bacteroides eggerthii strain JCM 12986 | 99 | **Bacteroidetes** |
| PC205 | Faecal | -ve | [Clostridium] glycyrrhizinilyticum strain ZM35 | 96 | **Firmicutes** |
| PC206 | Faecal | -ve | Dorea formicigenerans strain ASD2437 | 100 | **Firmicutes** |
| PC207 | Faecal | -ve | Blautia sp. Marseille-P3441 | 99 | **Firmicutes** |
| PC208 | Faecal | -ve | Bacteroides stercoris strain JCM 9496 | 99 | **Bacteroidetes** |
| PC209 | Faecal | -ve | Lachnospiraceae bacterium | 99 | **Firmicutes** |
| PC210 | Faecal | -ve | Bacteroides xylanisolvens strain A3 | 99 | **Bacteroidetes** |
| PC211 | Faecal | -ve | Bacteroides stercoris strain JCM 9496 | 99 | **Bacteroidetes** |
| PC212 | Faecal | -ve | Bacteroides xylanisolvens strain A3 | 100 | **Bacteroidetes** |
| PC213 | Faecal | -ve | Bacteroides vulgatus strain NMBE-5 | 99 | **Bacteroidetes** |
| PC214 | Faecal | -ve | Bacteroides vulgatus strain NMBE-5 | 99 | **Bacteroidetes** |
| PC215 | Faecal | -ve | Bacteroides vulgatus strain NMBE-5 | 99 | **Bacteroidetes** |
| PC216 | Faecal | -ve | Collinsella aerofaciens strain indica | 99 | **Actinobacteria** |
| PC217 | Faecal | -ve | Bacteroides vulgatus strain NMBE-5 | 99 | **Bacteroidetes** |
| PC218 | Faecal | -ve | [Clostridium] celerecrescens strain | 95 | **Firmicutes** |
| PC219 | Faecal | -ve | Clostridium hathewayi strain RCGLD1 | 99 | **Firmicutes** |
| PC220 | Faecal | -ve | Bacteroides fragilis YCH46 | 100 | **Bacteroidetes** |
| PC221 | Faecal | -ve | Bacteroides uniformis | 100 | **Bacteroidetes** |
| PC222 | Faecal | -ve | Bacteroides stercoris strain JCM 9496 | 99 | **Bacteroidetes** |
| PC223 | Faecal | -ve | Coprococcus eutactus | 99 | **Firmicutes** |
| PC224 | Faecal | -ve | Bacteroides xylanisolvens strain XB1A | 99 | **Bacteroidetes** |
| PC225 | Faecal | -ve | Bacteroides vulgatus ATCC 8482 | 99 | **Bacteroidetes** |
| PC226 | Faecal | -ve | Bacteroides eggerthii strain JCM 12986 | 99 | **Bacteroidetes** |
| PC227 | Faecal | -ve | Bacteroides uniformis | 100 | **Bacteroidetes** |
| PC228 | Faecal | -ve | Bacteroides stercoris strain NJ1305 | 99 | **Bacteroidetes** |
| PC229 | Faecal | -ve | Bacteroides uniformis | 100 | **Bacteroidetes** |
| PC230 | Faecal | -ve | Blautia sp. GD8 | 98 | **Firmicutes** |
| PC231 | Faecal | -ve | Coprococcus comes strain ATCC 27758 | 100 | **Firmicutes** |
| PC232 | Faecal | -ve | Bacteroides vulgatus strain NMBE-5 | 99 | **Bacteroidetes** |
| PC233 | Faecal | -ve | Blautia wexlerae strain AUH-JLD17 | 99 | **Firmicutes** |
| PC234 | Faecal | -ve | Bacteroides uniformis | 100 | **Bacteroidetes** |
| PC235 | Faecal | -ve | [Ruminococcus] torques | 100 | **Firmicutes** |
| PC236 | Faecal | -ve | Bacteroides uniformis | 100 | **Bacteroidetes** |
| PC237 | Faecal | -ve | Dorea longicatena strain LCR19 | 99 | **Firmicutes** |
| PC238 | Faecal | -ve | Bacteroides vulgatus strain NMBE-5 | 99 | **Bacteroidetes** |
| PC239 | Faecal | -ve | Bacteroides uniformis strain EBA25-2 | 100 | **Bacteroidetes** |
| PC241 | Biopsy | -ve | Tyzzerella sp. Marseille-P3062 | 99 | **Firmicutes** |
| PC242 | Biopsy | -ve | Bacteroides dorei CL03T12C01 | 100 | **Bacteroidetes** |
| PC243 | Biopsy | -ve | Tyzzerella sp. Marseille-P3062 | 99 | **Firmicutes** |
| PC244 | Biopsy | -ve | Coprobacillus cateniformis strain ASD2413 | 100 | **Firmicutes** |
| PC245 | Biopsy | -ve | Tyzzerella sp. Marseille-P3062 | 99 | **Firmicutes** |
| PC246 | Faecal | -ve | Parabacteroides distasonis strain ATCC 8503 | 99 | **Bacteroidetes** |
| PC247 | Faecal | -ve | Odoribacter splanchnicus strain NCTC10825 | 99 | **Bacteroidetes** |
| PC248 | Faecal | -ve | [Clostridium] lactatifermentans strain G17 | 95 | **Firmicutes** |
| PC249 | Faecal | -ve | Bacteroides uniformis strain EBA25-2 | 99 | **Bacteroidetes** |
| PC250 | Faecal | -ve | Bacteroides uniformis strain EBA25-2 | 100 | **Bacteroidetes** |
| PC251 | Faecal | -ve | Dorea longicatena strain LCR19 | 99 | **Firmicutes** |
| PC252 | Faecal | -ve | Roseburia sp. 1120 ? | 92 | **Firmicutes** |
| PC253 | Faecal | -ve | Bacteroides vulgatus strain NMBE-5 | 99 | **Bacteroidetes** |
| PC254 | Faecal | -ve | Bacteroides stercoris strain JCM 9496 | 99 | **Bacteroidetes** |
| PC255 | Faecal | -ve | Blautia wexlerae strain AUH-JLD17 | 100 | **Firmicutes** |
| PC256 | Faecal | -ve | Butyrate-producing bacterium SL7/1 | 100 | **Firmicutes** |
| PC257 | Faecal | -ve | Bacteroides vulgatus strain NMBE-5 | 99 | **Bacteroidetes** |
| PC258 | Faecal | -ve | [Ruminococcus] torques | 99 | **Firmicutes** |
| PC259 | Faecal | -ve | Blautia wexlerae strain AUH-JLD17 | 100 | **Firmicutes** |
| PC260 | Faecal | -ve | Blautia wexlerae strain AUH-JLD56 | 100 | **Firmicutes** |
| PC261 | Biopsy | -ve | Bacteroides stercoris, strain: baku-07 | 99 | **Bacteroidetes** |
| PC262 | Biopsy | -ve | [Eubacterium] hallii strain ATCC 27751 | 99 | **Firmicutes** |
| PC263 | Biopsy | -ve | Bacteroides dorei isolate HS2_L_2_B_045b | 100 | **Bacteroidetes** |
| PC264 | Biopsy | -ve | Bacteroides caccae strain ATCC 43185 | 99 | **Bacteroidetes** |
| PC265 | Biopsy | -ve | Faecalicatena sp. | 99 | **Firmicutes** |
| PC266 | Biopsy | -ve | Bacteroides uniformis | 100 | **Bacteroidetes** |
| PC267 | Biopsy | -ve | Peptoniphilus harei , isolate TID-12* | 100 | **Firmicutes** |
| PC268 | Biopsy | -ve | Bacteroides uniformis | 100 | **Bacteroidetes** |
| PC269 | Biopsy | -ve | Bacteroides vulgatus strain mpk | 100 | **Bacteroidetes** |
| PC270 | Biopsy | -ve | Parabacteroides distasonis | 99 | **Bacteroidetes** |
| PC271 | Biopsy | -ve | Blautia luti JCM 17040 | 100 | **Firmicutes** |
| PC272 | Biopsy | -ve | [Eubacterium] rectale strain ATCC 33656 | 100 | **Firmicutes** |
| PC273 | Biopsy | -ve | Blautia sp. GD8 | 99 | **Firmicutes** |
| PC274 | Biopsy | -ve | Bacteroides eggerthii strain JCM 12986 | 99 | **Bacteroidetes** |
| PC275 | Biopsy | -ve | Bacteroides vulgatus strain mpk | 100 | **Bacteroidetes** |
| PC276 | Biopsy | -ve | Butyrate-producing bacterium SL7/1 16S, Clostridium nexile | 99 | **Firmicutes** |
| PC277 | Biopsy | -ve | Bacteroides vulgatus strain mpk | 100 | **Bacteroidetes** |
| PC278 | Biopsy | -ve | Butyrate-producing bacterium SL7/1 | 100 | **Firmicutes** |
| PC279 | Biopsy | -ve | [Eubacterium] rectale strain ATCC 33656 | 100 | **Firmicutes** |
| PC280 | Biopsy | -ve | Bacteroides vulgatus strain mpk | 99 | **Bacteroidetes** |
| PC283 | Biopsy | -ve | [Clostridium] spiroforme strain JCM 1432 | 99 | **Firmicutes** |
| PC284 | Biopsy | -ve | Bacteroides dorei isolate HS2_L_2_B_045b | 100 | **Bacteroidetes** |
| PC285 | Biopsy | -ve | Butyrate-producing bacterium SS3/4 | 99 | **Firmicutes** |
| PC286 | Biopsy | -ve | Bacteroides vulgatus strain mpk | 100 | **Bacteroidetes** |
| PC287 | Biopsy | -ve | Dorea longicatena strain LCR19 | 100 | **Firmicutes** |
| PC288 | Biopsy | -ve | Lachnospiraceae bacterium G11* | 95 | **Firmicutes** |
| PC289 | Biopsy | -ve | Bacteroides uniformis strain JCM 5828 | 99 | **Bacteroidetes** |
| PC290 | Biopsy | -ve | Eubacterium ventriosum strain ATCC 27560* | 97 | **Firmicutes** |
| PC291 | Faecal | -ve | [Eubacterium] hallii strain ATCC 27751 | 99 | **Firmicutes** |
| PC292 | Faecal | -ve | Bacteroides stercoris strain JCM 9496 | 99 | **Bacteroidetes** |
| PC294 | Faecal | -ve | Bacteroides vulgatus strain mpk | 99 | **Bacteroidetes** |
| PC295 | Faecal | -ve | Bacteroides vulgatus strain mpk | 99 | **Bacteroidetes** |
| PC296 | Faecal | -ve | Bacteroides eggerthii strain JCM 12986 | 99 | **Bacteroidetes** |
| PC297 | Faecal | -ve | Faecalibacterium prausnitzii strain A2165 | 99 | **Firmicutes** |
| PC298 | Faecal | -ve | Faecalibacterium sp. strain F127-3 | 99 | **Firmicutes** |
| PC299 | Faecal | -ve | [Clostridium] spiroforme strain JCM 1432 | 99 | **Firmicutes** |
| PC300 | Faecal | -ve | Erysipelotrichaceae bacterium GAM147 | 99 | **Firmicutes** |
| PC301 | Faecal | -ve | Erysipelatoclostridium sp. SNUG30099 | 99 | **Firmicutes** |
| PC302 | Faecal | -ve | [Clostridium] spiroforme strain JCM 1432 | 99 | **Firmicutes** |
| PC303 | Faecal | -ve | [Clostridium] innocuum strain I46 | 99 | **Firmicutes** |
| PC304 | Faecal | -ve | Longicatena caecimuris strain DSM 29490 | 100 | **Firmicutes** |
| PC305 | Faecal | -ve | Bacteroides vulgatus strain mpk | 99 | **Bacteroidetes** |
| PC306 | Faecal | -ve | Erysipelatoclostridium sp. SNUG30099 | 99 | **Firmicutes** |
| PC307 | Faecal | -ve | Bacteroides eggerthii strain JCM 12986 | 99 | **Bacteroidetes** |
| PC308 | Faecal | -ve | Faecalibacterium prausnitzii | 99 | **Firmicutes** |
| PC309 | Faecal | -ve | Bacteroides vulgatus strain mpk | 100 | **Bacteroidetes** |
| PC311 | Faecal | -ve | Bacteroides uniformis | 100 | **Bacteroidetes** |
| PC313 | Faecal | -ve | Faecalibacterium prausnitzii | 99 | **Firmicutes** |
| PC314 | Faecal | -ve | Faecalibacterium prausnitzii strain A2165 | 99 | **Firmicutes** |
| PC315 | Faecal | -ve | Dorea longicatena strain LCR19 | 100 | **Firmicutes** |
| PC317 | Faecal | -ve | Dorea longicatena strain LCR19 | 100 | **Firmicutes** |
| PC318 | Faecal | -ve | Bacteroides eggerthii strain JCM 12986 | 99 | **Bacteroidetes** |
| PC319 | Faecal | -ve | [Eubacterium] rectale strain ATCC 33656 | 100 | **Firmicutes** |
| PC320 | Faecal | -ve | Bacteroides vulgatus strain mpk | 99 | **Bacteroidetes** |
| PC321 | Faecal | -ve | Parabacteroides distasonis | 99 | **Bacteroidetes** |
| PC322 | Faecal | -ve | [Ruminococcus] gnavus culture CCARM:9309 | 100 | **Firmicutes** |
| PC323 | Faecal | -ve | Faecalibacterium prausnitzii | 99 | **Firmicutes** |
| PC324 | Faecal | -ve | Faecalibacterium prausnitzii strain HTF-E | 99 | **Firmicutes** |
| PC325 | Faecal | -ve | Bacteroides vulgatus strain mpk | 99 | **Bacteroidetes** |
| PC326 | Faecal | -ve | Bacteroides vulgatus strain mpk | 99 | **Bacteroidetes** |
| PC327 | Faecal | -ve | Bacteroides vulgatus strain mpk | 99 | **Bacteroidetes** |
| PC328 | Faecal | -ve | Bacteroides stercoris strain JCM 9496 | 99 | **Bacteroidetes** |
| PC329 | Faecal | -ve | Bacteroides ovatus strain 30087 | 99 | **Bacteroidetes** |
| PC330 | Faecal | -ve | Dorea longicatena strain LCR19 | 99 | **Firmicutes** |
| PC331 | Faecal | -ve | Bacteroides vulgatus strain mpk | 100 | **Bacteroidetes** |
| PC332 | Faecal | -ve | Bacteroides xylanisolvens strain A3 | 100 | **Bacteroidetes** |
| PC333 | Faecal | -ve | Butyrate-producing bacterium PH05YA09 | 99 | **Firmicutes** |
| PC334 | Faecal | -ve | Bacteroides vulgatus strain mpk | 100 | **Bacteroidetes** |
| PC335 | Faecal | -ve | Roseburia intestinalis strain: JCM 17583 | 99 | **Firmicutes** |
| PC336 | Faecal | -ve | Bacteroides xylanisolvens strain XB1A | 100 | **Bacteroidetes** |
| PC337 | Faecal | -ve | Dorea longicatena strain 111-35 | 99 | **Firmicutes** |
| PC338 | Faecal | -ve | Blautia sp. GD8 | 99 | **Firmicutes** |
| PC339 | Faecal | -ve | Bacteroides stercoris strain JCM 9496 | 99 | **Bacteroidetes** |
| PC340 | Faecal | -ve | Bacteroides vulgatus strain mpk | 100 | **Bacteroidetes** |
| PC342 | Faecal | -ve | Coprococcus comes strain ATCC 27758 | 99 | **Firmicutes** |
| PC343 | Faecal | -ve | Bacteroides caccae strain ATCC 43185 | 99 | **Bacteroidetes** |
| PC344 | Faecal | -ve | Butyrate-producing bacterium PH05YA09 | 99 | **Firmicutes** |
| PC346 | Faecal | -ve | Bacteroides vulgatus strain mpk | 99 | **Bacteroidetes** |
| PC347 | Faecal | -ve | Bifidobacterium longum strain HBUAS55107 | 100 | **Actinobacteria** |
| PC348 | Faecal | -ve | Bifidobacterium adolescentis strain BBMN23 | 99 | **Actinobacteria** |
| PC349 | Faecal | -ve | Bacteroides vulgatus strain mpk | 100 | **Bacteroidetes** |
| PC350 | Faecal | -ve | Anaerostipes hadrus strain BPB5 | 98 | **Firmicutes** |
| PC351 | Biopsy | -ve | Butyrate-producing bacterium SS3/4 | 98 | **Firmicutes** |
| PC352 | Biopsy | -ve | Roseburia intestinalis strain: JCM 17583* | 99 | **Firmicutes** |
| PC353 | Biopsy | -ve | [Eubacterium] hallii strain ATCC 27751* | 99 | **Firmicutes** |
| PC354 | Biopsy | -ve | [Eubacterium] hallii strain ATCC 27751* | 99 | **Firmicutes** |
| PC355 | Biopsy | -ve | Megamonas sp. Marseille-P3344 | 99 | **Firmicutes** |
| PC356 | Biopsy | -ve | Catenibacterium mitsuokai strain: JCM 10606 | 98 | **Firmicutes** |
| PC357 | Biopsy | -ve | Collinsella aerofaciens strain: JCM 10793 | 100 | **Actinobacteria** |
| PC358 | Biopsy | -ve | Eubacterium ventriosum strain ATCC 27560 | 99 | **Firmicutes** |
| PC359 | Biopsy | -ve | Bifidobacterium bifidum strain PRI 1 | 100 | **Actinobacteria** |
| PC360 | Biopsy | -ve | Bacteroides uniformis |  | **Bacteroidetes** |
| PC361 | Biopsy | -ve | Megamonas sp. Marseille-P3344 | 99 | **Firmicutes** |
| PC362 | Biopsy | -ve | Bacteroides coprocola strain: M158 | 99 | **Bacteroidetes** |
| PC363 | Biopsy | -ve | Prevotella copri strain JCM 13464 | 98 | **Bacteroidetes** |
| PC364 | Biopsy | -ve | Prevotella copri strain ASD606 | 99 | **Bacteroidetes** |
| PC365 | Biopsy | -ve | Eubacterium ventriosum strain ATCC 27560 | 99 | **Firmicutes** |
| PC366 | Biopsy | -ve | Dorea longicatena Marseille-P2116 | 100 | **Firmicutes** |
| PC367 | Biopsy | -ve | Bacteroides dorei CL03T12C01 | 100 | **Bacteroidetes** |
| PC368 | Biopsy | -ve | Ruminococcus faecis strain Eg2 * | 100 | **Firmicutes** |
| PC369 | Biopsy | -ve | Prevotella copri strain ASD606 | 99 | **Bacteroidetes** |
| PC370 | Biopsy | -ve | Prevotella copri strain ASD606 | 99 | **Bacteroidetes** |
| PC371 | Biopsy | -ve | Megasphaera elsdenii S2 | 99 | **Firmicutes** |
| PC372 | Biopsy | -ve | [Ruminococcus] gnavus CCARM:9309* | 96 | **Firmicutes** |
| PC373 | Biopsy | -ve | Bacteroides thetaiotaomicron strain 82 | 100 | **Bacteroidetes** |
| PC374 | Biopsy | -ve | Prevotella copri strain ASD606 | 99 | **Bacteroidetes** |
| PC375 | Biopsy | -ve | Eubacterium rectale M104/1 | 99 | **Firmicutes** |
| PC376 | Biopsy | -ve | Eubacterium rectale M104/1 | 99 | **Firmicutes** |
| PC377 | Biopsy | -ve | Prevotella copri strain ASD606 | 99 | **Bacteroidetes** |
| PC378 | Biopsy | -ve | Blautia wexlerae strain AUH-JLD17 | 99 | **Firmicutes** |
| PC379 | Biopsy | -ve | Prevotella copri strain ASD606 | 99 | **Bacteroidetes** |
| PC380 | Biopsy | -ve | [Eubacterium] rectale strain ATCC 33656 | 99 | **Firmicutes** |
| PC381 | Biopsy | -ve | Ruminococcus faecis strain Eg2 | 100 | **Firmicutes** |
| PC382 | Biopsy | -ve | Hungatella sp. | 99 | **Firmicutes** |
| PC383 | Biopsy | -ve | Blautia obeum | 99 | **Firmicutes** |
| PC384 | Biopsy | -ve | Mitsuokella jalaludinii strain SB5 | 98 | **Firmicutes** |
| PC385 | Biopsy | -ve | Bifidobacterium adolescentis strain GMRS2 | 99 | **Actinobacteria** |
| PC386 | Biopsy | -ve | Prevotella copri strain ASD606 | 99 | **Bacteroidetes** |
| PC387 | Biopsy | -ve | Streptococcus salivarius strain ATCC 25975 | 100 | **Firmicutes** |
| PC388 | Biopsy | -ve | Mitsuokella jalaludinii strain SB5 | 98 | **Firmicutes** |
| PC389 | Biopsy | -ve | Blautia obeum | 100 | **Firmicutes** |
| PC390 | Biopsy | -ve | Faecalibacterium prausnitzii isolate S3G1* | 99 | **Firmicutes** |
| PC391 | Faecal | -ve | Collinsella aerofaciens strain: JCM 10793 | 100 | **Actinobacteria** |
| PC392 | Faecal | -ve | Roseburia faecis | 96 | **Firmicutes** |
| PC393 | Faecal | -ve | Bacteroides ovatus, strain: EFEL003 | 100 | **Bacteroidetes** |
| PC394 | Faecal | -ve | [Eubacterium] rectale strain ATCC 33656 | 100 | **Firmicutes** |
| PC395 | Faecal | -ve | Blautia obeum | 99 | **Firmicutes** |
| PC396 | Faecal | -ve | Coprococcus comes strain ATCC 27758 | 94 | **Firmicutes** |
| PC397 | Faecal | -ve | Prevotella copri strain ASD606 | 99 | **Bacteroidetes** |
| PC398 | Faecal | -ve | Fusicatenibacter saccharivorans TT-111 | 99 | **Firmicutes** |
| PC399 | Faecal | -ve | Longibaculum muris strain MT10-315-CC-1.2-2 | 93 | **Firmicutes** |
| PC400 | Faecal | -ve | Eubacterium ventriosum strain ATCC 27560 | 99 | **Firmicutes** |
| PC401 | Faecal | -ve | Butyrate-producing bacterium SS3/4 | 98 | **Firmicutes** |
| PC402 | Faecal | -ve | Erysipelatoclostridium sp. SNUG30370 | 99 | **Firmicutes** |
| PC403 | Faecal | -ve | Butyrate-producing bacterium GM2/1 | 97 | **Firmicutes** |
| PC404 | Faecal | -ve | Megasphaera elsdenii S2 | 99 | **Firmicutes** |
| PC405 | Faecal | -ve | Dorea longicatena strain ASD1256 | 100 | **Firmicutes** |
| PC406 | Faecal | -ve | Bacteroides plebeius strain M12 | 99 | **Bacteroidetes** |
| PC407 | Faecal | -ve | Catenibacterium mitsuokai strain: JCM 10606 | 99 | **Firmicutes** |
| PC408 | Faecal | -ve | Prevotella copri strain ASD606 | 99 | **Bacteroidetes** |
| PC409 | Faecal | -ve | Prevotella copri strain ASD606 | 99 | **Bacteroidetes** |
| PC410 | Faecal | -ve | Faecalibacterium sp. strain F20-3 | 99 | **Firmicutes** |
| PC411 | Faecal | -ve | Bacteroides ovatus, strain: EFEL003 | 100 | **Bacteroidetes** |
| PC412 | Faecal | -ve | Bacteroides coprocola, strain: M158 | 99 | **Bacteroidetes** |
| PC413 | Faecal | -ve | [Eubacterium] rectale strain ATCC 33656 | 100 | **Firmicutes** |
| PC415 | Faecal | -ve | Clostridiales bacterium Art 12/1 | 98 | **Firmicutes** |
| PC416 | Faecal | -ve | Blautia wexlerae strain AUH-JLD17 | 99 | **Firmicutes** |
| PC417 | Faecal | -ve | Prevotella copri strain ASD606 | 99 | **Bacteroidetes** |
| PC418 | Faecal | -ve | Dorea longicatena strain LCR19 | 100 | **Firmicutes** |
| PC420 | Faecal | -ve | Blautia wexlerae strain AUH-JLD17 | 99 | **Firmicutes** |
| PC421 | Faecal | -ve | Collinsella aerofaciens strain: JCM 10793 | 100 | **Actinobacteria** |
| PC422 | Faecal | -ve | Faecalicatena sp | 97 | **Firmicutes** |
| PC423 | Faecal | -ve | Prevotella copri strain ASD606 | 99 | **Bacteroidetes** |
| PC424 | Faecal | -ve | Roseburia inulinivorans, strain: JCM 17584 | 99 | **Firmicutes** |
| PC425 | Faecal | -ve | Bacteroides plebeius strain M12 | 99 | **Bacteroidetes** |
| PC426 | Faecal | -ve | Bacteroides caccae strain ATCC 43185 | 99 | **Bacteroidetes** |
| PC427 | Faecal | -ve | Blautia wexlerae strain AUH-JLD17 | 99 | **Firmicutes** |
| PC428 | Faecal | -ve | Prevotella copri strain ASD606 | 99 | **Bacteroidetes** |
| PC429 | Faecal | -ve | Megamonas sp. Marseille-P3344 | 99 | **Firmicutes** |
| PC430 | Faecal | -ve | Prevotella copri strain ASD606 | 99 | **Bacteroidetes** |
| PC432 | Biopsy | -ve | Mitsuokella jalaludinii, strain SB5 | 98 | **Firmicutes** |
| PC433 | Biopsy | -ve | Bacteroides plebeius strain M12 | 99 | **Bacteroidetes** |
| PC434 | Biopsy | -ve | Prevotella copri strain ASD606 | 99 | **Bacteroidetes** |
| PC435 | Biopsy | -ve | Anaerostipes hadrus strain BPB5 | 99 | **Firmicutes** |
| PC436 | Biopsy | -ve | Collinsella aerofaciens strain: JCM 10793 | 98 | **Actinobacteria** |
| PC438 | Biopsy | -ve | Prevotella copri strain ASD606 | 99 | **Bacteroidetes** |
| PC439 | Faecal | -ve | Megasphaera elsdenii S2 | 99 | **Firmicutes** |
| PC440 | Faecal | -ve | Prevotella copri strain ASD606 | 99 | **Bacteroidetes** |
| PC441 | Faecal | -ve | Prevotella copri strain ASD606 | 99 | **Bacteroidetes** |
| PC442 | Faecal | -ve | Blautia sp. GD8 | 99 | **Firmicutes** |
| PC443 | Biopsy | -ve | Bifidobacterium adolescentis strain BBMN23 | 100 | **Actinobacteria** |
| PC445 | Biopsy | -ve | Faecalibacterium prausnitzii strain CNCM_I_4575 | 99 | **Firmicutes** |
| PC447 | Biopsy | -ve | Collinsella aerofaciens strain D9-82 | 100 | **Actinobacteria** |
| PC448 | Biopsy | -ve | Faecalibacterium prausnitzii isolate S13A7 | 99 | **Firmicutes** |
| PC449 | Biopsy | -ve | Blautia sp. GD8 strain GD8 | 99 | **Firmicutes** |
| PC450 | Biopsy | -ve | Faecalibacterium prausnitzii strain CNCM_I_4575 | 99 | **Firmicutes** |
| PC452 | Biopsy | -ve | Faecalibacterium sp. strain F93-3 | 99 | **Firmicutes** |
| PC453 | Biopsy | -ve | Dorea longicatena strain: JCM 11232 | 100 | **Firmicutes** |
| PC454 | Biopsy | -ve | Collinsella aerofaciens strain D9-82 | 99 | **Actinobacteria** |
| PC455 | Biopsy | -ve | [Eubacterium] hallii | 99 | **Firmicutes** |
| PC456 | Biopsy | -ve | Anaerostipes hadrus strain BPB5 | 99 | **Firmicutes** |
| PC457 | Biopsy | -ve | Faecalibacterium sp. strain F109-2 | 99 | **Firmicutes** |
| PC458 | Biopsy | -ve | Prevotella sp. 109 | 96 | **Bacteroidetes** |
| PC459 | Biopsy | -ve | Bifidobacterium faecale strain HBUAS55087 | 100 | **Actinobacteria** |
| PC460 | Biopsy | -ve | Collinsella aerofaciens , strain: JCM 10793 | 100 | **Actinobacteria** |
| PC461 | Biopsy | -ve | Collinsella aerofaciens , strain: JCM 10793 | 100 | **Actinobacteria** |
| PC462 | Biopsy | -ve | Prevotella sp. 109 | 98 | **Bacteroidetes** |
| PC463 | Biopsy | -ve | Anaerostipes hadrus strain DSM 3319 | 100 | **Firmicutes** |
| PC464 | Biopsy | -ve | Bacteroides dorei CL03T12C01 | 99 | **Bacteroidetes** |
| PC465 | Biopsy | -ve | Blautia wexlerae strain AUH-JLD17 | 99 | **Firmicutes** |
| PC466 | Biopsy | -ve | Blautia obeum | 99 | **Firmicutes** |
| PC467 | Biopsy | -ve | Collinsella aerofaciens strain D9-82 | 100 | **Actinobacteria** |
| PC468 | Biopsy | -ve | [Eubacterium] hallii strain ATCC 27751 | 99 | **Firmicutes** |
| PC469 | Biopsy | -ve | Blautia wexlerae strain DSM 19850 | 99 | **Firmicutes** |
| PC471 | Biopsy | -ve | Blautia wexlerae strain AUH-JLD17 | 99 | **Firmicutes** |
| PC472 | Biopsy | -ve | Bacteroides clarus strain JCM 16067 | 100 | **Bacteroidetes** |
| PC473 | Biopsy | -ve | Anaerostipes hadrus strain 5/1/63FAA | 99 | **Firmicutes** |
| PC474 | Biopsy | -ve | Bacteroides dorei CL03T12C01 | 100 | **Bacteroidetes** |
| PC475 | Biopsy | -ve | [Eubacterium] hallii strain ATCC 27751 | 99 | **Firmicutes** |
| PC476 | Biopsy | -ve | Bacteroides kribbi strain R2F3-3-3 | 99 | **Bacteroidetes** |
| PC477 | Biopsy | -ve | Faecalibacterium sp. strain F22-2 | 99 | **Firmicutes** |
| PC478 | Biopsy | -ve | Bifidobacterium adolescentis strain BBMN23 | 100 | **Actinobacteria** |
| PC479 | Biopsy | -ve | Blautia wexlerae strain AUH-JLD17 | 99 | **Firmicutes** |
| PC480 | Biopsy | -ve | Butyrate-producing bacterium SS3/4 | 99 | **Firmicutes** |
| PC481 | Biopsy | -ve | Blautia wexlerae strain AUH-JLD17 | 100 | **Firmicutes** |
| PC482 | Biopsy | -ve | Blautia sp. GD8 | 100 | **Firmicutes** |
| PC483 | Faecal | -ve | Collinsella aerofaciens strain D9-82 | 99 | **Actinobacteria** |
| PC484 | Faecal | -ve | Collinsella aerofaciens strain D9-82 | 100 | **Actinobacteria** |
| PC485 | Faecal | -ve | Anaerostipes hadrus strain ASD1240 |  | **Firmicutes** |
| PC486 | Faecal | -ve | Erysipelatoclostridium sp. SNUG30099 |  | **Firmicutes** |
| PC487 | Faecal | -ve | Blautia wexlerae strain: JCM 17041 | 99 | **Firmicutes** |
| PC488 | Faecal | -ve | Faecalibacterium prausnitzii strain Indica | 99 | **Firmicutes** |
| PC489 | Faecal | -ve | Blautia sp. GD8 | 99 | **Firmicutes** |
| PC490 | Faecal | -ve | Faecalibacterium prausnitzii strain CNCM_I_4575 | 99 | **Firmicutes** |
| PC492 | Faecal | -ve | Ruminococcus bromii strain L2-63 | 99 | **Firmicutes** |
| PC493 | Faecal | -ve | Collinsella aerofaciens, strain: JCM 10793 | 100 | **Actinobacteria** |
| PC494 | Faecal | -ve | Anaerostipes hadrus strain DSM 3319 | 100 | **Firmicutes** |
| PC495 | Faecal | -ve | Bifidobacterium adolescentis strain FF065-1 | 99 | **Actinobacteria** |
| PC496 | Faecal | + ve | Enterococcus durans strain CAU9386 | 100 | **Firmicutes** |
| PC497 | Faecal | -ve | Butyrate-producing bacterium A2-207 | 99 | **Firmicutes** |
| PC498 | Faecal | -ve | Bacteroides finegoldii strain 199 | 99 | **Bacteroidetes** |
| PC499 | Faecal | -ve | Collinsella aerofaciens strain D9-82 | 100 | **Actinobacteria** |
| PC500 | Faecal | -ve | Bifidobacterium adolescentis strain BBMN23 | 100 | **Actinobacteria** |
| PC502 | Faecal | -ve | Faecalibacterium sp. strain F22-2 | 99 | **Firmicutes** |
| PC503 | Faecal | -ve | Bifidobacterium longum strain HBUAS55107 | 99 | **Actinobacteria** |
| PC504 | Faecal | -ve | Blautia wexlerae strain AUH-JLD17 | 99 | **Firmicutes** |
| PC505 | Faecal | -ve | Bacteroides caccae strain ATCC 43185 | 99 | **Bacteroidetes** |
| PC506 | Faecal | -ve | Collinsella aerofaciens strain D9-82 | 99 | **Actinobacteria** |
| PC507 | Faecal | -ve | Anaerostipes hadrus strain ASD1240 |  | **Firmicutes** |
| PC508 | Faecal | -ve | Anaerostipes hadrus strain DSM 3319 | 100 | **Firmicutes** |
| PC509 | Faecal | -ve | Anaerostipes hadrus strain BPB5 | 99 | **Firmicutes** |
| PC510 | Faecal | -ve | Prevotella sp. 109 | 98 | **Bacteroidetes** |
| PC511 | Faecal | -ve | Parabacteroides merdae | 98 | **Bacteroidetes** |
| PC512 | Faecal | -ve | Blautia wexlerae | 99 | **Firmicutes** |
| PC513 | Faecal | -ve | Anaerostipes hadrus strain BPB5 | 99 | **Firmicutes** |
| PC516 | Faecal | -ve | Blautia sp. Marseille-P2398 | 99 | **Firmicutes** |
| PC517 | Faecal | -ve | Prevotella sp. 109 | 98 | **Bacteroidetes** |
| PC518 | Faecal | -ve | Blautia sp. Marseille-P2398 | 99 | **Firmicutes** |
| PC519 | Faecal | -ve | Bifidobacterium adolescentis strain BBMN23 | 99 | **Actinobacteria** |
| PC520 | Faecal | -ve | Anaerostipes hadrus strain 5/1/63FAA | 99 | **Firmicutes** |
| PC521 | Faecal | -ve | Faecalibacterium sp. strain F109-2 | 99 | **Firmicutes** |
| PC522 | Faecal | -ve | Faecalibacterium prausnitzii strain CNCM_I_4575 | 99 | **Firmicutes** |
| PC524 | Biopsy | -ve | Faecalibacterium prausnitzii strain CNCM_I_4575 | 99 | **Firmicutes** |
| PC525 | Faecal | -ve | Bifidobacterium adolescentis strain BBMN23 | 99 | **Actinobacteria** |
| PC526 | Faecal | -ve | Faecalibacterium sp. strain F109-2 | 99 | **Firmicutes** |
| PC528 | Faecal | -ve | Faecalibacterium prausnitzii strain CNCM_I_4544 | 99 | **Firmicutes** |
