# Supplementary material for: Comparison of microbial signatures between paired faecal and rectal biopsy samples from healthy volunteers using next-generation sequencing and culturomics
Source: Microbiome. 2022 Oct 14;10:171. doi: 10.1186/s40168-022-01354-4 (PMC9563177; doi:10.1186/s40168-022-01354-4)
Supplement: Supplementary file 14 — Additional file 13: Figure S7. Detection of fermentation acids in media following growth of individual isolates in pure culture. Acid production split for biopsy wash (BW) and faecal (F) bacterial isolates. The upward arrow indicates fermentation acid production whilst the downward arrow represents consumption during bacterial growth in pure culture. Bacterial isolate numbers shown on the x-axis correspond to bacterial identities detailed in Additional file 12, Table S6. Data are averages of technical replicates from single cultures. [file 40168_2022_1354_MOESM13_ESM.docx]

**Additional file 13: Fig.S7.** Detection of fermentation acids in media following growth of individual isolates in pure culture. Acid production split for biopsy wash (BW) and faecal (F) bacterial isolates. The upward arrow indicates fermentation acid production whilst the downward arrow represents consumption during bacterial growth in pure culture. Bacterial isolate numbers shown on the x-axis correspond to bacterial identities detailed in Additional file 12, Table S6. Data are averages of technical replicates from single cultures.

**
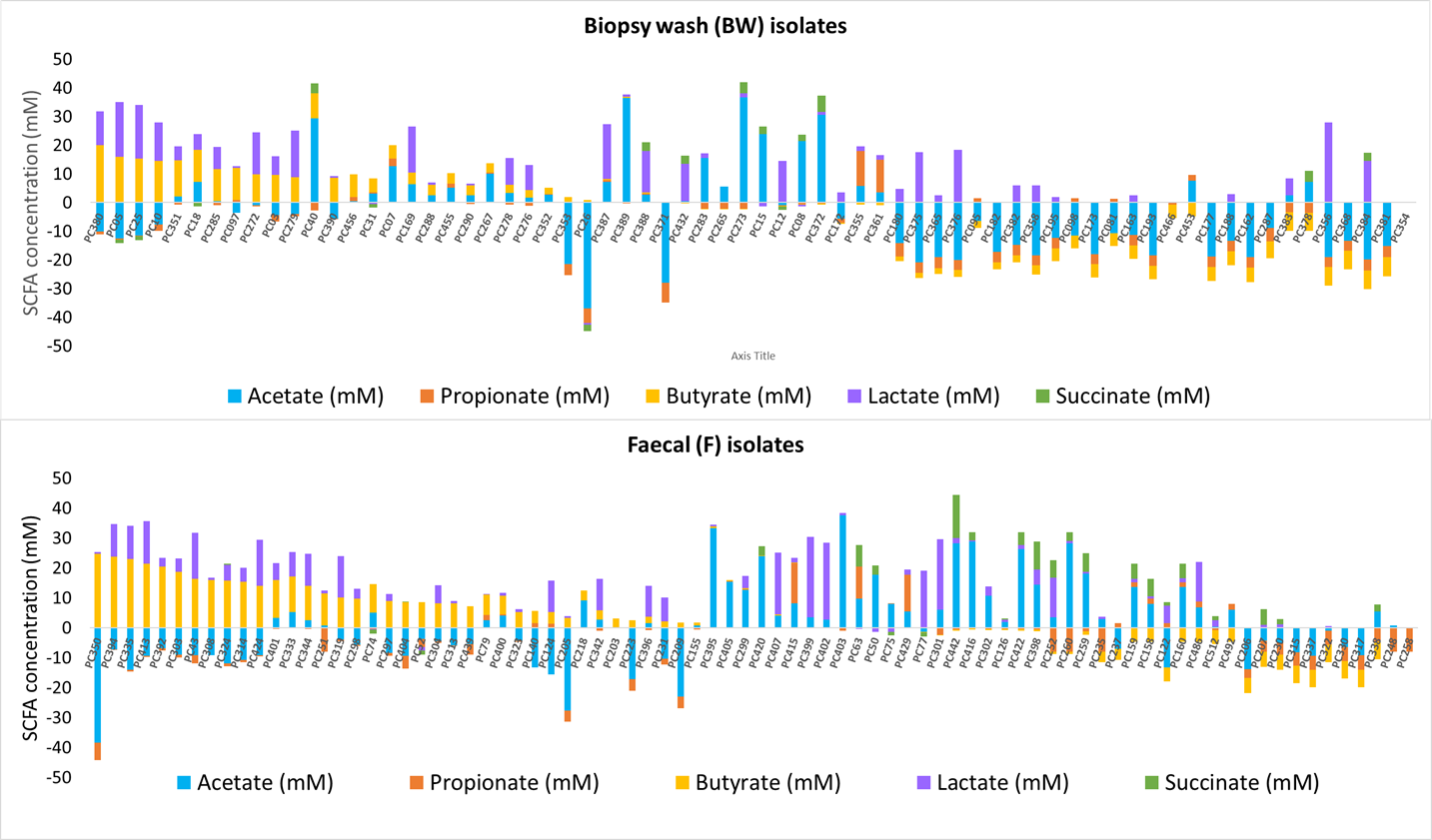
**
